# Structural insights into chromosome attachment to the nuclear envelope by an inner nuclear membrane protein Bqt4 in fission yeast

**DOI:** 10.1093/nar/gky1186

**Published:** 2018-11-20

**Authors:** Chunyi Hu, Haruna Inoue, Wenqi Sun, Yumiko Takeshita, Yaoguang Huang, Ying Xu, Junko Kanoh, Yong Chen

**Affiliations:** 1 State Key Laboratory of Molecular Biology, National Center for Protein Science Shanghai, Shanghai Science Research Center, CAS Center for Excellence in Molecular Cell Science, Shanghai Institute of Biochemistry and Cell Biology, Chinese Academy of Sciences; University of Chinese Academy of Sciences, 333 Haike Road, Shanghai 201210, China; 2 Institute for Protein Research, Osaka University, 3-2 Yamadaoka, Suita, Osaka 565-0871, Japan; 3 School of Life Science and Technology, Shanghai Tech University, 100 Haike Road, Shanghai 201210, P.R. China

## Abstract

The dynamic association of chromosomes with the nuclear envelope (NE) is essential for chromosome maintenance. *Schizosaccharomyces pombe* inner nuclear membrane protein Bqt4 plays a critical role in connecting telomeres to the NE, mainly through a direct interaction with the telomeric protein Rap1. Bqt4 also interacts with Lem2 for pericentric heterochromatin maintenance. How Bqt4 coordinates the interactions with different proteins to exert their functions is unclear. Here, we report the crystal structures of the N-terminal domain of Bqt4 in complexes with Bqt4-binding motifs from Rap1, Lem2, and Sad1. The structural, biochemical and cellular analyses reveal that the N-terminal domain of Bqt4 is a protein-interaction module that recognizes a consensus motif and plays essential roles in telomere-NE association and meiosis progression. Phosphorylation of Bqt4-interacting proteins may act as a switch to regulate these interactions during cell cycles. Our studies provide structural insights into the identification and regulation of Bqt4-mediated interactions.

## INTRODUCTION

Eukaryotic chromosomes are organized into various domains and localized to specific territories in the nucleus (1,2). In particular, heterochromatin regions are generally associated with the nuclear envelope (3–6). The striking difference between yeast and metazoan is that yeast cells do not possess lamins, which are major docking sites on the NE for metazoan chromosomes (7–9). Instead, the association between chromosomes and the NE in yeast cells are predominantly mediated by inner nuclear membrane (INM) proteins, which could regulate chromosome maintenance and genome stabilities (3,4).

Bqt4 is one of the most abundant INM proteins in fission yeast *Schizosaccharomyces pombe*, and plays crucial roles in chromosome-NE association by interacting with different protein partners. Bqt4 interacts directly with a telomere protein Rap1 to tether telomeres to the NE (10). In the absence of Bqt4, telomeres are dissociated from the NE, resulting in partial loss of meiotic telomere clustering, defective spore formation and decreased meiotic recombination frequency (10). Bqt4 also mediates the association between *mat* locus and the NE through a yet-to-be-identified molecular link (11). The specific tethering of these heterochromatin loci to the NE by Bqt4 provides an environment that prevents collisions between replication and transcription of these chromosome regions, although the underlying mechanism is not known (11). Moreover, Bqt4 interacts with another INM Lem2, and this interaction mobilizes Lem2 molecules around the NE to promote pericentric heterochromatin maintenance (11,12). The double-deletion of *lem2*^+^ and *bqt4*^+^ in *S. pombe* exhibits a synthetic lethal phenotype (13), underscoring the importance of cooperation between these two INM proteins in maintaining genome stability. How Bqt4 interacts with different proteins to exert specific functions in chromosome-NE association remains elusive.

Chromosomes positioning to the NE is dynamically regulated during the cell cycle. Telomeres are the best-characterized chromosomal sites that are dynamically associated with the NE (10,14). In *S. pombe*, telomeres are tethered to the NE during interphase, and are transiently dissociated from the NE during mitosis (15,16). The dissociation of telomeres from the NE is essential for accurate chromosome segregation (15). This dissociation relies on the mitosis-specific phosphorylation of Rap1-S513 by Cdc2 to disrupt Bqt4–Rap1 interaction (15,16). In meiotic prophase, the NE-tethered telomeres are clustered to the spindle pole body (SPB) to form a bouquet-like configuration (14,17). The formation of telomere bouquet is critical for efficient pairing and recombination of homologous chromosomes. Upon access into the first meiotic division, telomeres dissociate from the SPB in a concerted manner, referred as ‘telomere fireworks’ (18). However, the molecular mechanisms of spatial and temporal dynamics of the telomere-NE association are still poorly understood.

In the present study, we sought to elucidate how Bqt4 interact with different partner proteins, how these interactions contribute to the telomere-NE association, and how these interactions might be regulated by phosphorylation during cell cycles. We report the crystal structure of the N-terminal domain of Bqt4 in complex with the Bqt4-binding motif from Rap1. Structure-based mutational analyses demonstrate that proper connection between Bqt4 and Rap1 is required for telomere attachment to the NE, meiotic telomere clustering, and normal spore formation. Interestingly, the comparative structural analyses of Bqt4–Rap1 and Bqt4–Lem2 complexes reveal that the N-terminal domain of Bqt4 is a protein-interaction module recognizing a consensus motif, enabling us to identify another potential Bqt4-interacting protein Sad1. We also propose that phosphorylation of Rap1 can regulate the interaction between Bqt4 and Rap1, leading to dynamic regulation of telomere association with the NE. These data suggest that Bqt4 is an important platform protein with multiple dynamic binding partners ensuring proper chromosome-association with the NE.

## MATERIALS AND METHODS

### Protein expression and purification

Fission yeast bouquet protein Bqt4 fragments were cloned into a modified pET28b vector with a SUMO protein fused at the N terminus after the 6xHis tag. Bqt4 fragments were expressed in *Escherichia coli* BL21(DE3). After induction for 16 h with 0.2 mM IPTG at 16°C, the cells were harvested and resuspended in lysis buffer (50 mM Tris–HCl pH 8.0, 400 mM NaCl, 10% glycerol, 1 mM PMSF, 2 mM 2-mercaptoethanol, and home-made protease inhibitor cocktail). The 100× home-made protease inhibitor cocktail includes 100 mM PMSF, 100 mg/ml Benzamidine, 100 μg/ml Leupeptin, 100 μg/ml Aprotinin and 100 μg/ml Pepstatin. After sonication and centrifugation, the supernatant was mixed with Ni-NTA agarose beads (Qiagen) and rotated for 2 h at 4°C. Then Ulp1 protease was added at a molar ratio of 1:200 to remove the 6xHis and SUMO tag in the N-terminus of Bqt4 proteins. Bqt4 proteins were collected after on-beads Ulp1 digestion for 16 hours at 4°C, and was further purified by gel-filtration chromatography on Hiload Superdex200 column (GE Healthcare) equilibrated with buffer (25 mM Tris–HCl pH 8.0, 150 mM NaCl). The purified Bqt4 proteins were concentrated to 30 mg/ml and stored at −80°C.

The Rap1, Lem2 and Sad1 fragments were expressed in *Escherichia coli* BL21(DE3) using a modified pGEX-6P-1 vector with an N-terminal GST tag followed by 3C protease site(LEVLFQGP). After induction for 16 hours with 0.2 mM IPTG at 16°C, the cells were harvested and resuspended in lysis buffer (50 mM Tris–HCl pH 8.0, 400 mM NaCl, 10% glycerol, 1 mM PMSF, 5 mM DTT and home-made protease inhibitor cocktail). After sonication and centrifugation, the supernatant was mixed with glutathione-Sepharose 4B (GE) and rotated for 2 h at 4°C. Then 3C protease was added at a molar ratio of 1:100 to remove the GST tag. After on-beads 3C digestion, the proteins were further purified by gel-filtration chromatography on Hiload Superdex75 column (GE Healthcare) equilibrated with buffer (25 mM Tris–HCl pH 8.0, 150 mM NaCl). The fractions containing the peptides were collected and analyzed.

### Crystallization, data collection, and structure determination

The SUMO_21–92_-Bqt4_8–140_ fusion protein and Rap1_498–512_ were mixed at a molar ratio of 1:1.5 and the mixtures were used for crystallization. The complex was crystallized in 0.1 M HEPES sodium pH 7.5, 10% (v/v) 2-propanol, 20% (w/v) polyethylene glycol 4000. The SUMO_21–92_-Bqt4_8–140_ fusion protein and Lem2_261–279_ were mixed at a molar ratio of 1:1.5 and the complex was crystallized in 1600 mM sodium citrate tribasic. The Bqt4_2–140_ and Sad1_88–101_ were mixed at a molar ratio of 1:1.5 and the complex was crystallized in 2.4 M sodium malonate, pH7.0.

The diffraction data were collected at the beamline BL18U and BL19U1 of the Shanghai Synchrotron Radiation Facility, and processed using HKL3000 (19). The structure was determined by molecular replacement in Phaser (20) using apo Bqt4 (PDB: 5YBX) and SUMO (PDB: 1EUV) structures as searching models. The structure refinement was done in PHENIX package (21) and the model was manually built and refined in COOT (22).

### Yeast two-hybrid assay

Yeast cells growth and manipulation were done according to standard procedures. The yeast strain L40 (MATa his3Δ200 trp1-901 leu2-3112 ade2 LYS::(4lexAop-HIS3) URA3::(8lexAop-LacZ)GAL4) was used in this study. The yeast two-hybrid assays were performed with two plasmids pBTM116 (binding domain) and pACT2 (activation domain). The colonies were selected on –Leu –Trp plates. The β-galactosidase activities were measured by liquid assay according to the standard manual.

### Isothermal titration calorimetry

The equilibrium dissociation constants of interactions were determined by using an ITC200 calorimeter (MicroCal). The enthalpies of binding between Rap1_479–527_ (1000–1500 μM) and Bqt_2–140_ (100–150 μM) were measured at 20°C in 20 mM Tris–HCl (pH 8.0) and 150 mM NaCl. Two independent experiments were performed for every interaction described here. ITC data were subsequently analyzed and fit with one binding site model using Origin 7 software (OriginLab) with blank injections of peptides into buffer subtracted from the experimental titrations prior to data analysis.

### GST pull-down assays

For the pull-downs of GST-tagged Rap1 with Bqt4, 10 μl of glutathione-Sepharose 4B beads were suspended with 50 μl of binding buffer (25 mM Tris, pH 8.0 and 100 mM NaCl, 10% glycerol). 30 μg of GST-tagged Rap1 and 20 μg purified Bqt4 proteins were added into the beads suspension and incubated at 4°C for 40 min. The beads were washed four times with 150 μl of binding buffer. The beads-bound proteins were eluted by 50 μl of SDS-PAGE sample loading buffer. All the input controls and beads-bound samples were analyzed with SDS-PAGE.

For the pull-down of Rap1 from fission yeast cell lysate by GST-Bqt4, GST or GST-Bqt4 proteins bound to glutathione beads were mixed with *S. pombe* cell extracts in TNE buffer (40 mM Tris–HCl, pH7.5, 150 mM NaCl, 5 mM EDTA, 50 mM NaF, 20 mM β-glycerophosphate) at 4°C for 2 h and washed with TNE buffer. The protein samples were analyzed with SDS-PAGE, followed by immunoblotting and Coomassie Brilliant Blue (CBB) gel staining.

### Fluorescence polarization competition assay

Lem2_BBM_ was labeled with FITC fluorescence dye using EZLabel Protein FITC Labeling Kit (Cat. No. K832-5, Biovision). For measuring the binding between Bqt4_NTD_ and Lem2_BBM_, 100nM FITC-labeled Lem2_BBM_ peptides were mixed with Bqt4_NTD_ proteins in a serial of concentrations from 20nM to 50 μM. For FP competition assays, the FITC-labeled Lem2_BBM_ peptides were pre-mixed with Bqt4_NTD_ at 4°C for 1 h. Serial dilutions of competitive peptides (from 10 nM to 200 μM) were added into the pre-formed Bqt4_NTD_–Lem2_BBM_ complex in 384-well plates, The final volume was brought up to 30 μl with dilution buffer (25 mM Tris–HCl, pH 8.0, 150 mM NaCl, 10% glycerol) and plates were incubated for 1 h with gentle shaking. The fluorescence polarization values in 384-well black plates were measured using Synergy Neo Multi-Mode Reader (Bio-Tek) at 25°C. The excitation wavelength was 485 nm and the emission was detected at 528 nm. Fluorescence was quantitated with GEN 5 software and date was analyzed with Prism 6 (GraphPad Software, San Diego, CA, USA). IC_50_ (half maximal inhibitory concentration) values were determined by nonlinear regression fitting of the competition curves.

### Strains, media and general techniques for *S. pombe*

The *S. pombe* strains used in this study are listed in Supplementary Table S1. Growth media, basic genetic and biochemical techniques were as described previously (23,24).

### Fluorescence microscopy

Optical section images were obtained at focus intervals of 0.3 μm using a DeltaVision microscopy system (Applied Precision, Issaquah, USA) and deconvolved by a 3D deconvolution method using softWoRx v5.5 software (25).

### Southern blotting

Apa1-digested genomic DNA was separated by conventional agarose gel electrophoresis and subjected to Southern blotting. For the telomere probe, telomeric DNA was excised from pNSU70.

### Measurement of the distance between the telomere and the NE

Telomeres, the NE, and microtubules were visualized with Taz1-mCherry, Ish1-GFP and GFP-Atb2, respectively. The distance between the telomeres and the NE was measured using a DeltaVision microscope system (Applied Precision, Issaquah, USA) as previously described (15). Briefly, we selected only telomeres that showed the brightest Taz1-mCherry signals, which correspond to telomeres on chromosomes I and/or II, and measured the distances from the center of the telomere signal to the middle of the NE signal. For the measurement of the distances in interphase cells, we chose cells of various cell lengths to avoid bias.

### Antibodies

For the detection of Rap1, GFP-tagged Bqt4, Flag tag, and Cdc2, we used anti-Rap1 (26), anti-GFP (JL-8; Clontech, Mountain View, USA), anti-Flag (F3165; Sigma) and anti-PSTAIR (P7962; Sigma), respectively.

## RESULTS

### The overall structure of Bqt4_NTD_-Rap1_BBM_ complex

**Figure 1.**
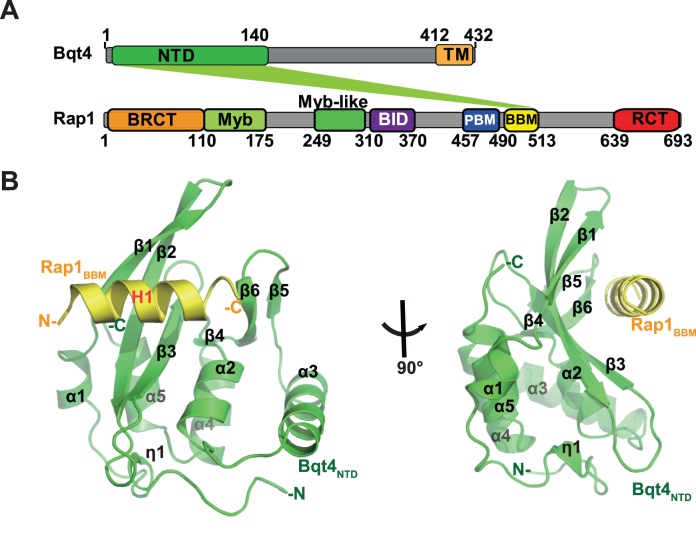
Structure of the Bqt4_NTD_-Rap1_BBM_ complex. (**A**) Domain organization of the spBqt4 and spRap1. NTD, Bqt4 N-Terminal Domain; TM, Transmembrane helix; BRCT, BRCA1 C-Terminal domain; Myb, Myb-type DNA-binding domain; BID, Bqt1/2-Interacting Domain; PBM, Poz1-Binding Motif; BBM, Bqt4-Binding Motif; RCT, Rap1 C-Terminal domain. (**B**) Two orthogonal views of the overall structure of the Bqt4_NTD_-Rap1_BBM_ complex. The core region of Bqt4_NTD_ is colored in green, and N-terminal extension of Bqt4_NTD_ is colored in cyan.

The N-terminal region of Bqt4 (Bqt4_2–180_) has been shown to interact with a Rap1 fragment (residues 220–606) (10,15). Thus we generated a series of Rap1 constructs covering residues 220–606 to evaluate their interactions with Bqt4_2–180_ by isothermal titration calorimetry (ITC) and GST pull-down assays (Supplementary Figure S1A-E). A minimal Rap1 fragment consisting of residues 490–513 was both necessary and sufficient for interaction with Bqt4_2–140_ and Bqt4_2–180_ (Supplementary Figure S1A-E). Hereafter, we refer to Rap1_490–513_ as the Bqt4-binding motif of Rap1 (Rap1_BBM_) and Bqt4_2–140_ as the N-terminal domain of Bqt4 (Bqt4_NTD_) (Figure 1A) unless stated otherwise.

After extensive screening, we determined the structure of Bqt4_NTD_-Rap1_BBM_ complex consisting of Sumo-fused Bqt4_8–140_ and Rap1_498–512_ at 2.7Å resolution (Supplementary Figure S2A, B and Table 1). There are three extra residues (GPL) at the N-terminus of Rap1 after removing GST fusion tag by the 3C protease. The final refined model included Sumo residues 24–92, Bqt4 residues 8–139, and Rap1 residues 498–509 with the N-terminal GPL residues. There were two Bqt4_NTD_-Rap1_BBM_ complexes in one asymmetric unit. The two complexes share almost identical structures with a root-mean-square deviation (RMSD) of 0.73 Å for 129 equivalent C_α_ pairs. The conformational differences in two complexes were observed in the loop regions connecting β strands, especially for the L_23_ loop between β2 and β3 (Supplementary Figure S2C).

**Table 1.** Data collection and refinement statistics for SUMO-Bqt4_NTD_-Rap1_BBM_ complex, SUMO-Bqt4_NTD_-Lem2_BBM_, and Bqt4_NTD_-Sad1_BBM_ complexes

	SUMO-Bqt4_NTD_-Rap1_BBM_	SUMO-Bqt4_NTD_-Lem2_BBM_	Bqt4_NTD_-Sad1_BBM_
**Data collection**			
Space group	*P*1	*P*2_1_2_1_2_1_	*P*6_3_22
Cell dimensions			
*a, b, c* (Å)	32.154, 51.293, 79.669	43.868, 53.128, 109.566	133.951,133.951,87.394
α, β, γ (°)	94.920, 100.556, 105.001	90, 90, 90	90, 90, 120
Wavelength (Å)	0.97853	0.97775	0.97775
Resolution (Å)	50-2.7	50-1.57	50-2.6
*R*_merge_	0.089(0.324)	0.076 (0.264)	0.115(0.712)
*I*/σ*I*	11.7 (2.4)	51.7 (9.0)	29.8 (3.3)
Completeness (%)	97.9 (94.8)	99.9 (98.6)	100 (100)
Redundancy	3.5 (2.9)	12.9(11.6)	12.5 (12.8)
**Refinement**			
Resolution (Å)	39.06–2.70	27.39–1.57	43.8–2.6
No. reflections	12761	36534	14641
*R*_work_/*R*_free_ (%)	20.2/24.9	18.9/21.4	17.5/20.4
No. atoms			
Protein	3149	1644	1063
Peptide	228	139	132
Water	33	294	26
*B*-factors (Å^2^)			
Protein	53.6	20.8	60.5
Peptide	55.5	36.4	74.8
Water	43.4	31.4	63.0
R.m.s deviations			
Bond lengths (Å)	0.002	0.006	0.009
Bond angles (°)	0.481	1.167	1.036

^a^Values in parentheses are for highest-resolution shell.

The Bqt4_NTD_ comprises six α helices and six β strands arranged in a compact α/β fold (Figure 1B). Rap1_BBM_ forms an α-helix and spans on top of the β-sheet composed of strands β1–β4 of Bqt4_NTD_ (Figure 1B). The overall structure of Bqt4_NTD_ is similar to that of apo-Bqt4 (manuscript submitted), with an RMSD value of 0.69 Å for 129 C_α_ pairs (Supplementary Figure S2D). Notably, the loop between α3 and α4 was rearranged to form two new β strands (β5 and β6) upon Rap1_BBM_ binding (Supplementary Figure S2D).

### The interface between Bqt4_NTD_ and Rap1_BBM_

**Figure 2.**
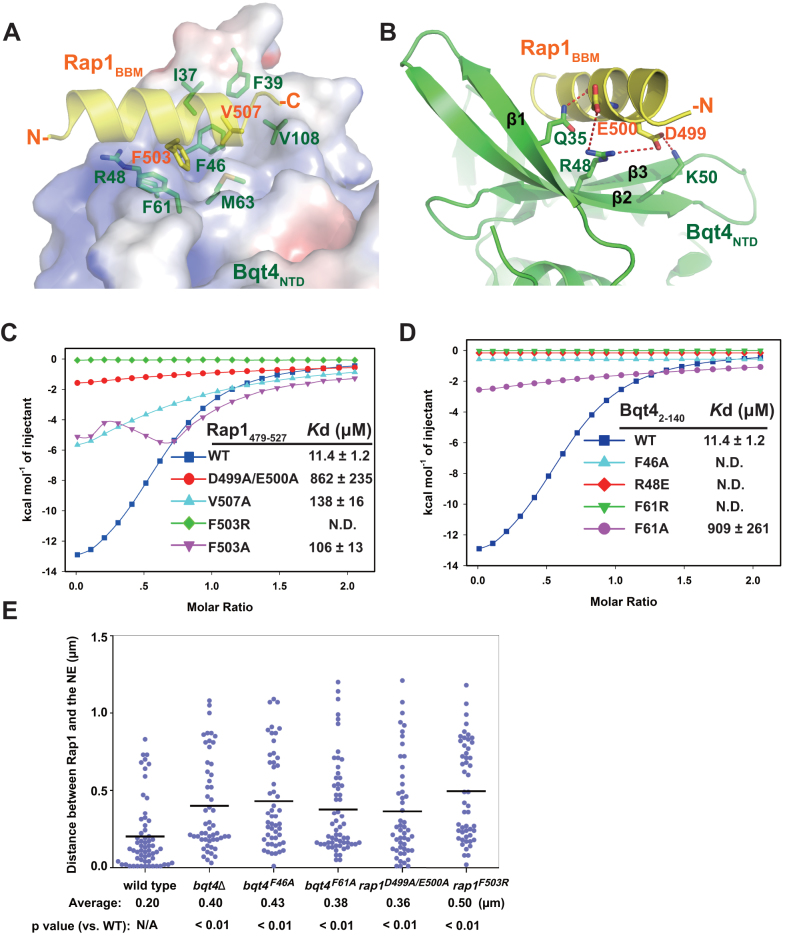
The interface between Bqt4_NTD_ and Rap1_BBM_. (**A**) Details of hydrophobic interactions between Bqt4_NTD_ and Rap1_BBM_. Bqt4_NTD_ surface is colored according to its electrostatic potential (positive potential, blue; negative potential, red). The interaction residues are presented as ball-and-stick models. Bqt4 residues are colored in green and Rap1 residues are colored in yellow. (**B**) Details of electrostatic and hydrogen-bonding interactions between Bqt4_NTD_ and Rap1_BBM_. Salt-bridges and hydrogen bonding interactions are shown as dashed magenta lines. (**C** and **D**) ITC measurement of the interaction between Bqt4_NTD_ and Rap1_479–527_. The fitting curves of the titration data and the derived dissociation constant (*K*_d_) value ± SD were shown. All the mutations decreased or abolished the interaction between Bqt4_NTD_ and Rap1_BBM_. The same curves for the wild-type Bqt4_NTD_-Rap1_479–527_ were used in these two panels. (**E**) Rap1 is dissociated from the NE in the *bqt4* and *rap1* mutants. Scatterplots showed the distances between Rap1-mCherry and the NE (Ish1-GFP) from more than 50 cells. Horizontal bars in the graph indicate the average distances. *P*, Mann–Whitney U test versus wild-type.

The interaction between Bqt4_NTD_ and Rap1_BBM_ is mediated by a combination of hydrophobic and electrostatic interactions (Figure 2A and B). The C-terminal half of Rap1_BBM_ helix is hydrophobic and contacts a hydrophobic pocket of Bqt4 (Figure 2A). Two hydrophobic residues of Rap1 (F503 and V507) form a hydrophobic core that fits snugly into a shallow groove formed by β1–β4 of Bqt4_NTD_ (Figure 2A). Rap1^F503^ is sandwiched between Bqt4^F46^ and Bqt4^F61^ by π-π stacking interactions. Rap1^V507^ makes intimate contacts with a hydrophobic pocket formed by M63, V108, I37, and F39 of Bqt4. To corroborate the structural analysis, we used ITC and GST pull-down assays to examine whether mutations of these interface residues of Bqt4 and Rap1 could weaken or disrupt the interaction between Bqt4_2–140_ and Rap1_479–527_. Consistent with the structural model, Rap1 mutations (F503A and V507A) severely destabilized the Bqt4–Rap1 complex, and the Rap1^F503R^ mutation completely abolished the interaction between Bqt4 and Rap1 (Figure 2C, Supplementary Figure S3A and C). On the other side of the interface, Bqt4 mutations (F46A and F61A) also disrupted the Bqt4–Rap1 interaction (Figure 2D, Supplementary Figure S3B and C), which suggests that these hydrophobic contacts are crucial for Rap1 binding with Bqt4.

Besides the hydrophobic contacts, the N-terminal half of Rap1_BBM_ helix is rich in acidic residues, and sits on a basic patch of Bqt4_NTD_ through electrostatic interactions (Figure 2A and B). In particular, Rap1^D499^ coordinates two salt bridges with Bqt4^R48^ and Bqt4^K50^. Rap1^E500^ engages in a salt-bridge interaction with Bqt4^R48^, and also forms a hydrogen bond with Bqt4^Q35^ (Figure 2B). Mutations of these charged residues (Bqt4^R48E^ and Rap1^D499A/E500A^) severely impaired the binding of Rap1 to Bqt4 as shown by ITC assays and GST-pull-down assays (Figure 2C, D, Supplementary Figure S3A and C), reinforcing the importance of the electrostatic interaction network between Bqt4_NTD_ and Rap1_BBM_.

To further examine the Bqt4–Rap1 interaction *in vivo*, we checked whether mutations of the interface residues affect cellular localization of Rap1 and Bqt4 by microscopic observation of living cells. GFP-fused Bqt4 exclusively localized to the NE. Rap1-binding-deficient mutations of Bqt4 did not affect the NE-localization at all, suggesting that Rap1-binding is dispensable for Bqt4 localization (Supplementary Figure S4A). It is expected since Bqt4 has a C-terminal transmembrane helix determining its inner nuclear membrane localization. We then examined the subcellular localization of mCherry-fused Rap1. Wild-type Rap1 was localized close to the NE during the interphase of mitotically growing cells (Supplementary Figure S4B). In contrast, both *rap1* mutations (D499A/E500A and F503R) and *bqt4* mutations (F46A and F61A) caused dissociation of Rap1 from NE in interphase cells (Figure 2E and Supplementary Figure S4B). It suggests that these mutations disrupted the Bqt4–Rap1 interaction *in vivo*, and subsequently detached Rap1 from the NE. Overall, our mutagenesis analyses confirmed that the interface observed in the minimal Bqt4–Rap1 complex was important for the interaction between Bqt4 and Rap1 both *in vitro* and *in vivo*.

### Functional study of the Bqt4–Rap1 interaction

We further investigated the *in vivo* functional importance of the interaction between Bqt4 and Rap1. Effects of mutations deficient in the Bqt4–Rap1 interaction were examined in a variety of cellular phenotypes. All the Bqt4 or Rap1 mutant proteins were expressed at near wild-type levels in *S. pombe*, suggesting that these mutations did not interfere with protein expression or protein stability (Supplementary Figure S5A). Rap1 is a critical negative regulator of telomere length (27–29), and deletion of *rap1* from yeast cells resulted in long and highly heterogeneous telomere DNA (Supplementary Figure S5B). However, neither *bqt4* nor *rap1* mutations caused any telomere length defect (Supplementary Figure S5B), confirming the previous notion that the Bqt4–Rap1 interaction is dispensable for the telomere maintenance (30). Moreover, the disruption of Bqt4–Rap1 interactions did not show any sensitivity to various stress conditions (Supplementary Figure S5C), indicating that the Bqt4–Rap1 interaction is not essential for cell viability.

**Figure 3.**
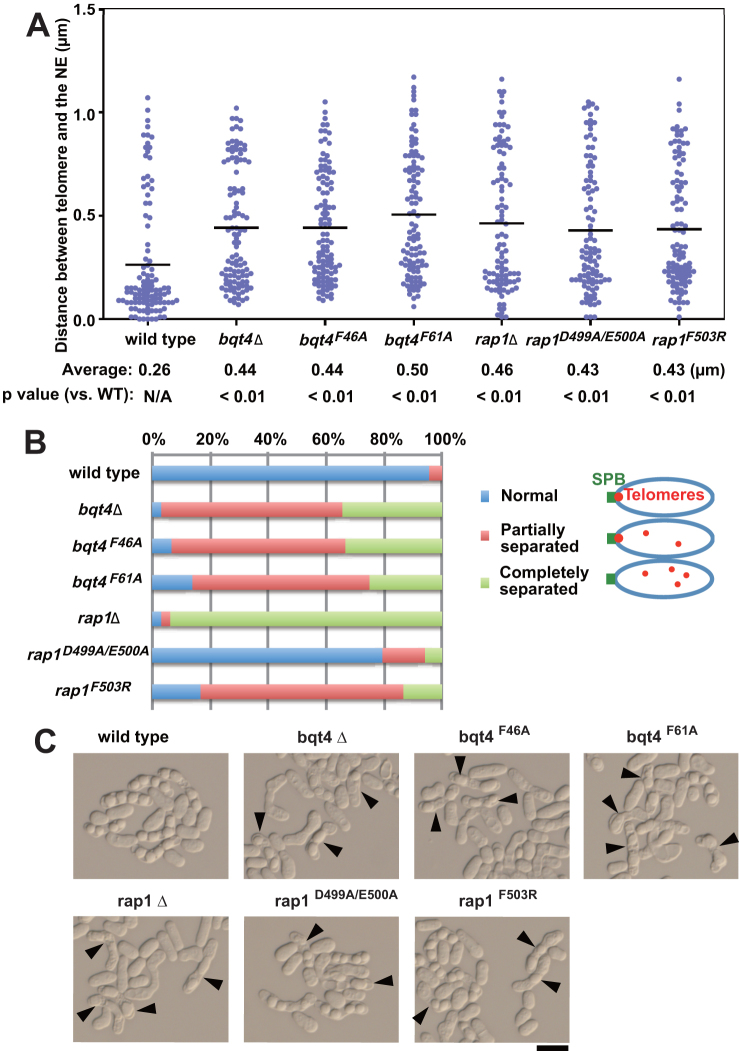
Functional studies of the Bqt4–Rap1 interaction. (**A**) Scatterplots showed the distances between telomeres and the NE during interphase in mitotically growing cells. Telomeres, the NE and microtubules were visualized with Taz1-mCherry, Ish1-GFP, and GFP-Atb2, respectively. More than 100 nuclei were analyzed for each strain. Horizontal bars in the graph indicated the average distances. *P*, Mann-Whitney U test versus wild-type. (**B**) Meiotic telomere clustering is defective in the *bqt4* and *rap1* mutants, as shown by the proportion of three types of telomere positions in meiotic horsetail stage. More than 30 cells were analyzed for each strain. Blue, all telomeres are clustered to the SPB; red, a portion of telomeres are dissociated from the SPB; green, all telomeres are dissociated from the SPB. (**C**) Representative DIC images showing spore formation. Homothallic haploid cells were incubated on MEA plates at 28°C for 2 days. Arrowheads indicate the abnormal cells with abnormal spore numbers (1, 2 or 3 spores). The scale bar indicates 10 micrometers.

In the absence of Bqt4, telomeres failed to associate with the NE in vegetative cells and consequently were unable to cluster to the SPB in meiotic prophase, resulting in obvious defects in spore formation (10). Consistent with the previous report (15), the microscopic analyses showed that deletion of *bqt4* or *rap1* resulted in the increased distance between the telomeres and the NE in interphase cells (Figure 3A). Similar to *bqt4Δ* and *rap1Δ* cells, the *rap1* mutations (*rap1^D499A/E500A^* and *rap1^F503R^*) and *bqt4* mutations (*bqt4^F46A^* and *bqt4^F61A^*) that abolished Bqt4–Rap1 interactions significantly increased the distance between the telomeres and the NE (Figure 3A), confirming that Bqt4–Rap1 interaction plays a crucial role in telomere attachment to the NE. We then checked the telomere clustering to the SPB in the *bqt4* and *rap1* mutants during meiotic prophase. The *bqt4Δ* and *rap1Δ* strains completely lost the association of telomeres to the SPB (Figure 3B and Supplementary Figure S6). All the *bqt4* and *rap1* mutants except for *rap1^D499A/E500A^* showed dramatic defects in telomere clustering to SPB (Figure 3B), further confirming that the stable Bqt4–Rap1 interaction is important for meiotic telomere clustering to the SPB. As a result of defective meiotic telomere clustering, the *bqt4^F46A^, bqt4^F61A^*, and *rap1^F503R^* mutants generated abnormal spore numbers instead of normal four spores during sporulation (Figure 3C). The sporulation defects of these mutants were similar to that of *bqt4*Δ and *rap1*Δ strains (Figure 3C). Collectively, our mutational studies revealed that the Bqt4–Rap1 interaction is essential for telomere attachment to the NE, meiotic telomere clustering to the SPB, and normal sporulation.

### The structure of Bqt4–Lem2 complex

**Figure 4.**
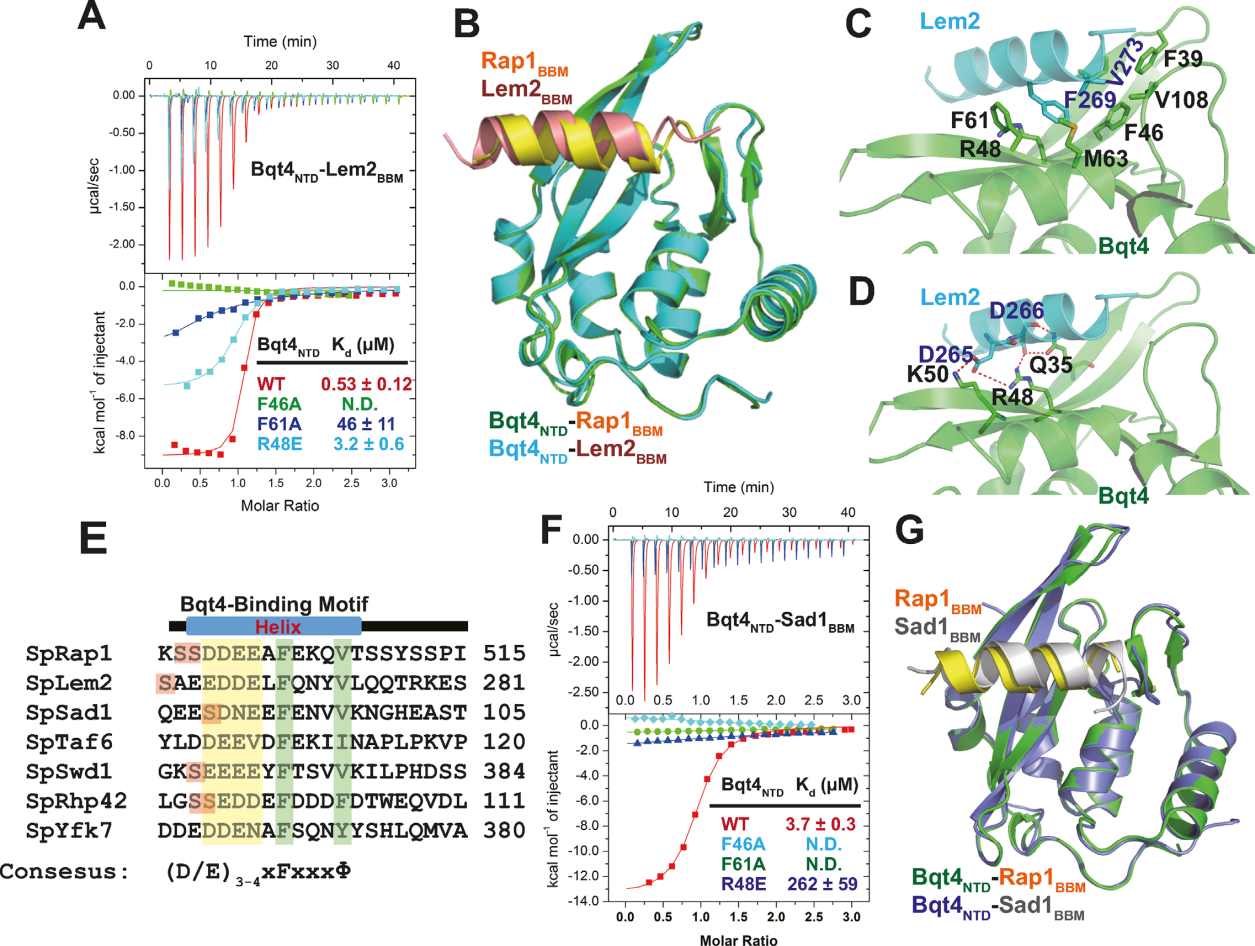
Bqt4_NTD_ recognizes a conserved motif. (**A**) ITC measurements of the interaction between Bqt4_NTD_ and Lem2_BBM_. The upper panel is the heat change upon titration of Rap1 into Bqt4 and the lower panel is binding isotherm fit to one binding site model. (**B**) Superimposition of Bqt4_NTD_–Lem2_BBM_ and Bqt4_NTD_–Rap1_BBM_ structures. Bqt4_NTD_ in Bqt4_NTD_–Lem2_BBM_ complex is colored in cyan and Lem2_BBM_ is colored in red. Bqt4_NTD_ in Bqt4_NTD_–Rap1_BBM_ complex is colored in green and Rap1_BBM_ is colored in yellow. (**C**) Details of hydrophobic interactions between Bqt4_NTD_ and Lem2_BBM_. (**D**) Details of electrostatic and hydrogen-bonding interactions between Bqt4_NTD_ and Rap1_BBM_. (**E**) A conserved Bqt4-binding motif found in some fission yeast proteins. Φ stands for hydrophobic residues. (**F**) ITC measurements of the interaction between Bqt4_NTD_ and Sad1_BBM_. Sad1_BBM_ bound to Bqt4_NTD_^WT^ but not with Bqt4_NTD_^F46A^, Bqt4_NTD_^F46A^, and Bqt4_NTD_^R48E^. (**G**) Superimposition of Bqt4_NTD_–Sad1_BBM_ and Bqt4_NTD_–Rap1_BBM_ structures showed almost identical configurations of these two complexes.

Besides interaction with Rap1, Bqt4 was recently reported to directly interact with Lem2 to regulate pericentric heterochromatin maintenance (11,12). Here we used yeast two-hybrid, GST pull-down, and ITC assays to characterize the interaction between Bqt4 and Lem2. The data showed that Bqt4_NTD_ interacted with a short fragment of Lem2 (residues 261–279), referred to as Lem2_BBM_ (Supplementary Figure S7A-D). ITC assays confirmed that Lem2_BBM_ could interact with Bqt4_NTD_ with a *K*_d_ around 0.53 μM (Figure 4A and Supplementary Figure S7D). To understand how Bqt4_NTD_ recognizes Lem2_BBM_, we determined the crystal structure of the Bqt4_NTD_-Lem2_BBM_ (Figure 4B and Table 1). Surprisingly, the complex structure of Bqt4_NTD_-Lem2_BBM_ exhibits remarkable similarity with Bqt4_NTD_–Rap1_BBM_ (Figure 4B). Lem2_BBM_ adopts the same helical configuration as Rap1_BBM_, and interacts with Bqt4 through the corresponding surface contacts as seen in the Bqt4_NTD_–Rap1_BBM_ complex (Figure 4B). All the hydrophobic contacts and electrostatic interactions are conserved in both complexes (Figure 4C and D). For example, Lem2^F269^ is sandwiched by Bqt4^F46^ and Bqt4^F61^, in the same fashion as Rap1^F503^ (Figure 4C). Bqt4^R48^ coordinates two salt bridges with Lem2^D265^ and Lem2^D266^ (Figure 4D). Mutations of Bqt4^F46A^, Bqt4^F61A^, and Bqt4^R48A^ decreased the interaction with Lem2_BBM_ to different degrees (Figure 4A), indicating that the observed interface in the crystal structure is essential for the interaction between Bqt4_NTD_ and Lem2_BBM_. These Bqt4 mutations did not affect Lem2 protein levels *in vivo* (Supplementary Figure S7E), suggesting that Bqt4–Lem2 interaction is not critical for expression or stability of Lem2. Taken together, these data reveal that Bqt4 recognizes a short motif of Lem2 in a similar binding mode as that observed in the Bqt4–Rap1 complex.

### Bqt4_NTD_ recognizes a consensus motif

Notably, Rap1_BBM_ and Lem2_BBM_ share a common sequence feature: three to four consecutive acidic residues followed by x-F-x-x-x-Φ (Φ stands for hydrophobic residues) (Figure 4E). Having identified a conserved Bqt4-binding motif in Rap1 and Lem2, we were interested to know whether other proteins in *S. pombe* contain this conserved motif and may interact with Bqt4 in the same manner. Accordingly, we immediately identified several *S. pombe* proteins containing this conserved sequence motif by ScanProsite (31). These proteins including Sad1, a spindle pole body-associated protein; Taf6, a subunit in transcription initiation factor TFIID; Swd1, a component in H3K4 methyltransferase SET1 complex; Rhp42, a DNA-repair protein; and YFK7, an E3 ubiquitin ligase (Figure 4E). Among these proteins, Sad1 has been shown to interact with Bqt1, and mediates the attachment of telomeres to the NE during meiosis (14). A 14-residue fragment of Sad1 (residues 88–101), referred to as Sad1_BBM_, can bind Bqt4_NTD_ with the dissociate constant of 3.7 μM, as shown by ITC assays (Figure 4F and Supplementary Figure S7F). We further determined the crystal structure of Bqt4_NTD_-Sad1_BBM_ complex (Table 1), and found that Bqt4_NTD_–Sad1_BBM_ structure is virtually same as Bqt4_NTD_–Rap1_BBM_ and Bqt4_NTD_–Lem2_BBM_ structures (Figure 4G). Sad1_BBM_ interacts with Bqt4_NTD_ in the same manner as that in Bqt4_NTD_–Rap1_BBM_ and Bqt4_NTD_–Lem2_BBM_ complexes. Mutations of the critical Bqt4 residues, including F46, F61, and R48, did not affect Sad1 stability, but severely impeded the interaction with Sad1_BBM_ (Figure 4F and Supplementary Figure S7G). Overall, our data suggest that Bqt4_NTD_ is a protein-interaction module that recognizes a consensus motif.

### Rap1 and Lem2 bind Bqt4 competitively

**Figure 5.**
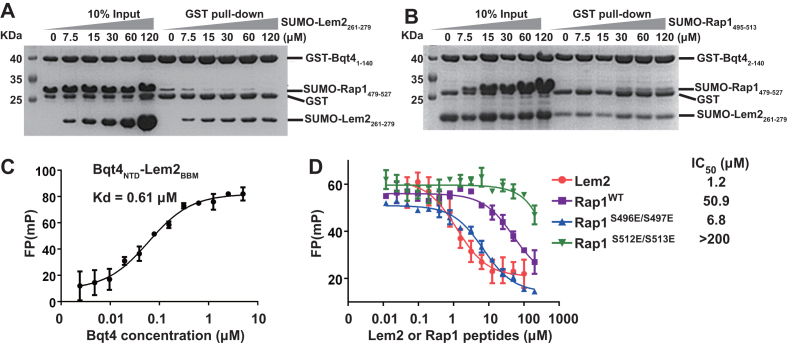
Rap1 and Lem2 bind Bqt4 competitively. (**A**) *In vitro* GST pull-down assays examining interactions between GST-Bqt4_NTD_ and SUMO-Rap1_479–527_ in the presence of different concentrations of Lem2_261–279_. 15μM GST-Bqt4_NTD_ and 20μM SUMO-Rap1_479–527_ were incubated for 30 minutes and then titrated with SUMO-Lem2_261–279_ from 7.5 to 120 μM. 15 μM SUMO-Lem2_261–279_ can effectively dissociate SUMO-Rap1_479–527_ from Bqt4_NTD_. (**B**) In vitro GST pull-down assays examining interactions between GST-Bqt4_NTD_ and SUMO-Lem2_261–279_ in the presence of different concentrations of Rap1_479–527_. 15μM GST-Bqt4_NTD_ and 20μM SUMO-Lem2_261–279_ were incubated for 30 minutes and then titrated with SUMO-Rap1_479–527_ from 7.5 to 120 μM. Even the highest concentration of Rap1_479–527_ cannot dissociate Lem2_BBM_ from Bqt4_NTD_. (**C**) FP assays showed that fluorescent FITC-labeled Lem2_BBM_ bound Bqt4_NTD_. Each point was represented as mean ± s.d. (*n* = 3). (**D**) Competitive FP binding curves of Lem2, Rap1^WT^, Rap1^S512E/S513E^, and Rap1^S496E/S497E^ binding to the pre-formed fluorescent Bqt4_NTD_-Lem2_BBM_ complex. The IC_50_ value was determined by nonlinear regression fitting of the competition curves. Each point was represented as mean ± s.d. (*n* = 3).

Because all the proteins containing Bqt4-binding-motifs bind to the same molecular surface of Bqt4_NTD_, the association of these factors to Bqt4_NTD_ is mutually exclusive and competitive. To test this idea, we performed the *in vitro* competition pull-down assays. The pre-formed Bqt4_NTD_–Rap1_479–527_ complex could be dissociated by the addition of Lem2_BBM_ in a dosage-dependent manner (Figure 5A). On the contrary, the pre-formed Bqt4_NTD_-Lem2_BBM_ complex could be hardly disrupted by the titration of Rap1, as the Bqt4–Lem2 interaction was 20-fold stronger than the Bqt4–Rap1 interaction (Figure 5B). We further utilized fluorescence polarization (FP) assays to quantitatively characterize the competition between Rap1 and Lem2 for binding with Bqt4_NTD_. The FP assay showed the dissociation constant (*K*_d_) between Bqt4_NTD_ and the fluorescent FITC-labeled Lem2_BBM_ to be 0.61 μM, similar as that determined by ITC (*K*_d_ = 0.53 μM) (Figure 5C). The complex composed of Bqt4_NTD_ and FITC-labeled Lem2_BBM_ were then titrated with unlabeled Rap1 or Lem2 peptides, resulting in the decrease of the FP signal due to the displacement of FITC-labeled Lem2_BBM_. The value of IC_50_ (half maximal inhibitory concentration) at which concentration half complex was dissociated could reflect the competition ability of unlabeled peptides. The Lem2_261–279_ peptide (IC_50_ = 1.2 μM) was 40-fold more efficient than Rap1_479–527_ peptide (IC_50_ = 50.9 μM) in competing for binding with Bqt4_NTD_ (Figure 5D). Thus, we conclude that Rap1 and Lem2 bind to the same pocket of Bqt4 in a competitive manner.

### Regulation of the Bqt4–Rap1 interaction by phosphorylation

**Figure 6.**
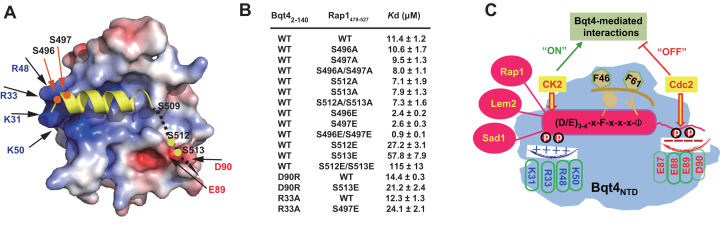
Regulation of Bqt4–Rap1 interaction by Rap1 phosphorylation. (**A**) Serine residues on Rap1 are phosphorylated during mitosis and meiosis. Bqt4_NTD_ is shown in surface representation and colored according to its electrostatic potential (positive potential, blue; negative potential, red). Rap1_BBM_ is shown in yellow. A dashed black line indicates one possible path of the absent C-terminal tail of Rap1_BBM_. (**B**) The dissociation constants of Bqt4_NTD_-Rap1_479–527_ interactions determined by ITC assays. (**C**) The model of Bqt4-mediated interactions and the phosphorylation switches.

After elucidating that Bqt4 could interact with a panel of proteins using the same interaction mode, an intriguing issue is how the cell dynamically coordinates different proteins associated with Bqt4. The structures of Bqt4_NTD_ complexes with the Bqt4-binding motif from Rap1 implied a potential molecular mechanism for the regulation of complex formation or dissociation (Figure 6A).

Rap1 is heavily phosphorylated during both mitosis and meiosis (15,16,32). Several phosphorylation sites are within or in the vicinity of the Bqt4-binding motif, including S497, S509, S510, S512 and S513 (15,32). These serine residues do not mediate direct interaction with Bqt4, as mutations of serine residues have minimal effects on binding affinities between Bqt4 and Rap1 (Figure 6B and Supplementary Figure S8A). Previous studies showed that phosphorylation of Rap1^S513^ by Cdc2 weakened the Bqt4–Rap1 interaction and dissociated telomere from the NE in the M phase (15). The ITC measurements confirmed that the Rap1^S513E^ mutation, which mimics constitutive serine phosphorylation, resulted in a 5-fold decrease in the binding affinity with Bqt4_NTD_ (Figure 6B and Supplementary Figure S8B). Although Rap1^S513^ is absent from the current Bqt4_NTD_-Rap1_BBM_ structural model, calculation of the electrostatic potential of Bqt4_NTD_ shows that the C-terminal tail of Rap1_BBM_ helix could bind to an acidic patch of Bqt4 (_87_EEED_90_) through one possible path indicated in Figure 6A. Thus, a negatively-charged phosphate group deposited on the C-terminal tail of Rap1_BBM_ may weaken the binding with Bqt4. To support this notion, phosphorylation-mimic mutation of another nearby serine residue (Rap1^S512^) also decreased the interaction with Bqt4, and a double mutation of Rap1 (Rap1^S512E/S513E^) further reduced the binding affinity by 10-fold compared with wild-type Rap1 (Figure 6B and Supplementary Figure S8B). The loss of the Lem2-competition ability of Rap1^S512E/S513E^ also suggested the decreased interaction between Bqt4 and Rap1^S512E/S513E^ (Figure 5D). Additionally, substitution of Bqt4^D90^ into a positively-charged arginine increased the binding affinity with Rap1^S513E^ (Figure 6B and Supplementary Figure S8C), confirming this hypothetical configuration of the C-terminal tail of Rap1_BBM_. These data indicate that phosphorylation of the C-terminal tail of Rap1_BBM_ by Cdc2 can attenuate the interaction between Bqt4 and Rap1, and may serve as an ‘off’ switch for Bqt4–Rap1 interaction (Figure 6C).

In contrast with C-terminal region of Rap1_BBM_, the N-terminal region of Rap1_BBM_ is localized onto a basic patch of Bqt4_NTD_, including K31, R33, R48, and K50 residues from Bqt4_NTD_ (Figure 6A). Thus, we speculated that certain phosphorylation of the N-terminal region of Rap1_BBM_ could enhance the interaction with Bqt4. Primary sequence examination suggests that the N-terminal region of Rap1_BBM_ contains two potential Casein Kinase 2 (CK2) sites (S[D/E] or Sxx[D/E]): S496 and S497. Indeed, phosphorylation of S497 has been detected in *S. pombe* at meiosis I stage (32). Both Rap1^S496E^ and Rap1^S497E^ mutants resulted in increased binding affinity between Bqt4_2–140_ and Rap1_479–527_ (Figure 6B and Supplementary Figure S8B). The double mutant of Rap1_479–527_ (S496E/S497E) had 10-fold higher binding affinity with Bqt4_2–410_ than wild-type Rap1. The increased binding affinity between Rap1^S496E/S497E^ and Bqt4 was also confirmed by the increased FP competition ability of this mutation (Figure 5D). Accordingly, the mutation on the basic patch of Bqt4 (R33A), which may sense the phosphorylation of Rap1 S496/S497, reversed the enhanced interaction with Rap1^S497E^ (Figure 6B and Supplementary Figure S8C). Therefore, we propose that the phosphorylation of the N-terminal region of Rap1_BBM_ can augment the interaction between Bqt4 and Rap1, and may serve as an ‘on’ switch for the Bqt4–Rap1 interaction (Figure 6C).

## DISCUSSION

In the present work, we determined the crystal structures of Bqt4_NTD_ in complexes with Bqt4-binding-motifs from Rap1, Lem2, and Sad1. Although these structures are obtained from the minimal interacting-domains, they do represent the dominant interaction interfaces of the full-length complexes. First, the mutations of the crucial Bqt4 and Rap1 residues observed in our structures disrupted the interaction between Bqt4 and full-length Rap1 (Supplementary Figure S3C). Second, all the functional analyses were based on the point mutations of full-length Bqt4 and Rap1 proteins. These cellular analyses proved that the mutations disrupted Bqt4–Rap1 interactions and consequently led to similar phenotypes as seen in *bqt4-null* and *rap1-null* cells. At last, these interaction motifs from Rap1, Lem2, and Sad1 are localized in the flexible regions, and predicted to be independent on other domains in these proteins. However, at this stage, we cannot exclude the possibility that BBM-nearby regions may regulate Bqt4_NTD_-BBM interactions. For example, the Bqt4-binding-motif of Rap1 (residues 490–513 of Rap1) is very close to Poz1-binding-motif (residues 467–491 of Rap1). Whether Poz1-binding induces any conformational change of Rap1 to modulate its Bqt4-binding activity needs further investigation.

Our results provide a general framework for the identification and understanding of the Bqt4-mediated interactions (Figure 6C). The identification of the consensus motif will facilitate us to search for more putative Bqt4-interacting partners in the future. Here we have verified that Sad1 contains a Bqt4-binding-motif and binds Bqt4_NTD_ using the same interaction mode as observed in Bqt4–Rap1 and Bqt4–Lem2 complexes. Sad1 is a SUN-domain-containing inner-nuclear-membrane protein, and interacts with a KASH-domain-containing outer-nuclear-membrane Kms1 to form a trans-nuclear-membrane complex which is referred to as a linker of nucleoskeleton and cytoskeleton complex (LINC) (14). Sad1 is localized to spindle pole body (SPB) and plays important roles in meiotic telomere bouquet formation (14). Determining whether Sad1 interacts with Bqt4 *in vivo* and revealing the functional significance of this interaction merit further study.

We have provided evidence that Lem2 and Rap1 competitively bind Bqt4, by competitive GST pull-down and FP assays *in vitro* (Figure 6A and B). Although we lack the direct evidence whether the competition occurs *in vivo*, a recent report indicated that Rap1 and Lem2 might compete with each other for Bqt4-binding *in vivo* (11). This report revealed that Bqt4 colocalized with Lem2 and Bqt4 colocalized with telomeres (possibly through the Bqt4–Rap1 interaction), but Lem2 showed a markedly lower degree of colocalization with telomeres (11). It is consistent with our hypothesis that the binding of Rap1 and Lem2 to Bqt4 is mutually exclusive and competitive. However, whether the competition happens in the cells also depends on the relative concentration of these proteins. According to a previous proteomic study (33), there are 4467 Bqt4, 249 Rap1, 934 Lem2 and 2369 Sad1 molecules in a single vegetative cell. Bqt4 is more abundant than other proteins. Theoretically, there are enough Bqt4 molecules to bind all interaction partners. How Bqt4 molecules coordinate the interactions with different Bqt4-binding-motif containing proteins *in vivo* remains to be elucidated.

It has been shown that the Bqt4–Rap1 interaction could be modulated by Rap1 phosphorylation (15). Here we provide structural and biochemical evidences that the phosphorylations on the N-terminus and C-terminus of Rap1_BBM_ could increase and decrease the interaction between Rap1 and Bqt4, respectively (Figure 6B). We propose that the N-terminus of Rap1_BBM_ contained putative CK2-recognition sites and could be phosphorylated by CK2 to enhance the interaction with Bqt4. We are currently investigating whether Bqt4 can be phosphorylated by CK2 *in vivo* and how the dynamic phosphorylation of Rap1 turns ‘on’ or ‘off’ its interaction with Bqt4 during cell cycles. Notably, the putative CK2 phosphorylation sites are also present in other potential Bqt4-binding proteins (Figure 4E, red-shadow residues). For example, S88 of Sad1, at the N-terminus of Sad1_BBM_, had been reported to be phosphorylated in three phosphoproteomics analyses (34–36). Thus, it might be a common feature for Bqt4-mediated interactions, which are regulated by cell-cycle-dependent or DNA-damage-response-dependent phosphorylation events (Figure 6C). It indicates that Bqt4 functions not only as the NE-docking platform for various proteins, but also as coordinators to orchestrate complicated interaction networks in a precisely controlled manner.

The major function of Bqt4 currently known is to anchor telomeres to the NE, which affect the progression of meiosis and mitosis (10,15,16). Substantial evidence indicates the possibility that Bqt4 might have additional roles at the non-telomere regions. There are more than ∼4000 copies of Bqt4 and only ∼250 copies of Rap1 in one cell (33,37), strongly indicating that Bqt4 might have additional non-telomeric interaction partners other than Rap1. Recently, another inner nuclear membrane protein Lem2 was found to interact with Bqt4 to promote pericentric heterochromatin maintenance and maintain genome stability (11–13). Here we have identified some putative Bqt4-interaction proteins, and these proteins have a variety of cellular functions, including histone modification, ubiquitination, DNA repair, and transcription initiation, suggesting that Bqt4 might have much broader functions than currently known. Overall, these data will stimulate further investigation on non-telomeric functions of Bqt4.

## DATA AVAILABILITY

Coordinate and structure factor have been deposited in the Protein Data Bank under accession codes 5YC2 (Bqt4_NTD_-Rap1_BBM_), 5YCA (Bqt4_NTD_-Lem2_BBM_) and 6A6W (Bqt4_NTD_-Sad1_BBM_).

## Supplementary Material

Supplementary Data
