# Correction to ‘Structural insights into chromosome attachment to the nuclear envelope by an inner nuclear membrane protein Bqt4 in fission yeast’

**DOI:** 10.1093/nar/gkab1181

**Published:** 2021-11-24

**Authors:** Chunyi Hu, Haruna Inoue, Wenqi Sun, Yumiko Takeshita, Yaoguang Huang, Ying Xu, Junko Kanoh, Yong Chen

**Affiliations:** State Key Laboratory of Molecular Biology, National Center for Protein Science Shanghai, Shanghai Science Research Center, CAS Center for Excellence in Molecular Cell Science, Shanghai Institute of Biochemistry and Cell Biology, Chinese Academy of Sciences; University of Chinese Academy of Sciences, 333 Haike Road, Shanghai 201210, China; Institute for Protein Research, Osaka University, 3-2 Yamadaoka, Suita, Osaka 565-0871, Japan; State Key Laboratory of Molecular Biology, National Center for Protein Science Shanghai, Shanghai Science Research Center, CAS Center for Excellence in Molecular Cell Science, Shanghai Institute of Biochemistry and Cell Biology, Chinese Academy of Sciences; University of Chinese Academy of Sciences, 333 Haike Road, Shanghai 201210, China; School of Life Science and Technology, Shanghai Tech University, 100 Haike Road, Shanghai 201210, P.R. China; Institute for Protein Research, Osaka University, 3-2 Yamadaoka, Suita, Osaka 565-0871, Japan; State Key Laboratory of Molecular Biology, National Center for Protein Science Shanghai, Shanghai Science Research Center, CAS Center for Excellence in Molecular Cell Science, Shanghai Institute of Biochemistry and Cell Biology, Chinese Academy of Sciences; University of Chinese Academy of Sciences, 333 Haike Road, Shanghai 201210, China; State Key Laboratory of Molecular Biology, National Center for Protein Science Shanghai, Shanghai Science Research Center, CAS Center for Excellence in Molecular Cell Science, Shanghai Institute of Biochemistry and Cell Biology, Chinese Academy of Sciences; University of Chinese Academy of Sciences, 333 Haike Road, Shanghai 201210, China; Institute for Protein Research, Osaka University, 3-2 Yamadaoka, Suita, Osaka 565-0871, Japan; State Key Laboratory of Molecular Biology, National Center for Protein Science Shanghai, Shanghai Science Research Center, CAS Center for Excellence in Molecular Cell Science, Shanghai Institute of Biochemistry and Cell Biology, Chinese Academy of Sciences; University of Chinese Academy of Sciences, 333 Haike Road, Shanghai 201210, China; School of Life Science and Technology, Shanghai Tech University, 100 Haike Road, Shanghai 201210, P.R. China

Nucleic Acids Research, Volume 47, Issue 3, 20 February 2019, Pages 1573–1584, https://doi.org/10.1093/nar/gky1186

Wenqi Sun is a joint graduate student of Shanghai Tech University and University of Chinese Academy of Sciences.

This change in the affiliations does not affect the results, discussion and conclusions presented in the article.

