# Supplementary material for: Structural insights into chromosome attachment to the nuclear envelope by an inner nuclear membrane protein Bqt4 in fission yeast
Source: Nucleic Acids Res. 2018 Nov 20;47(3):1573–84. doi: 10.1093/nar/gky1186 (PMC6379675; doi:10.1093/nar/gky1186)
Supplement: Supplementary Data [file gky1186_supplemental_files.pdf]

Structural insights into chromosome attachment to the nuclear envelope by an inner nuclear membrane protein Bqt4 in fission yeast

Chunyi Hu[1#], Haruna Inoue[3#], Wenqi Sun[2#], Yumiko Takeshita[3], Yaoguang Huang[1], Ying Xu[1], Junko Kanoh[3] and Yong Chen[1,2]

[1]State Key Laboratory of Molecular Biology, National Center for Protein Science Shanghai, Shanghai Science Research Center, CAS Center for Excellence in Molecular Cell Science, Shanghai Institute of Biochemistry and Cell Biology, Chinese Academy of Sciences; University of Chinese Academy of Sciences, 333 Haike Road, Shanghai 201210, China.

[2]School of Life Science and Technology, Shanghai Tech University, 100 Haike Road, Shanghai 201210, P. R. China

[3]Institute for Protein Research, Osaka University, 3-2 Yamadaoka, Suita, Osaka 565-0871, Japan

# These authors contributed equally to this work

Correspondence should be addressed to Y.C. (yongchen@sibcb.ac.cn) or J.K. (jkanoh@protein.osaka-u.ac.jp).

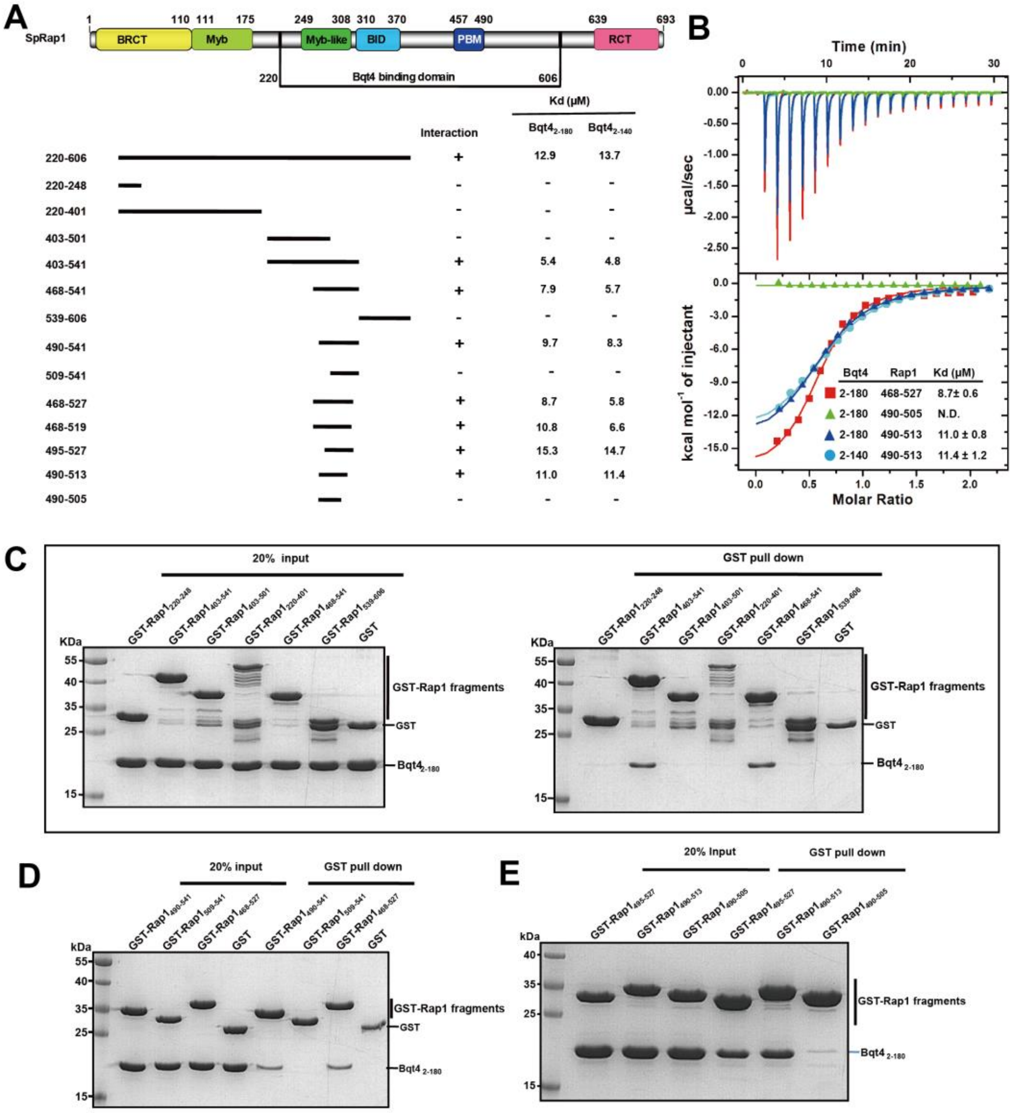

Supplementary Figure 1. Mapping the interaction domains between Bqt4 and Rap1.

A. Summary of Rap1constructs used for mapping the minimum Bqt4-binding motif of Rap1. The Kd values were determined by Isothermal titration calorimetry (ITC) assays.

B. Representative ITC data for the binding of Rap1 fragments to Bqt4. The upper panel is the heat change upon titration of Rap1 into Bqt4 and the lower panel is binding isotherm fit to a single binding site.

C, D and E. GST pull-down assays identified the minimum Rap1 region responsible for binding to $Bqt4_{2-180}$. Different fragments of Rap1 were fused with GST and used to pull down $Bqt4_{2-180}$. The input controls were shown as 20% of total proteins used for pull down assays. GST pull-down samples were eluted by SDS-PAGE loading buffer.

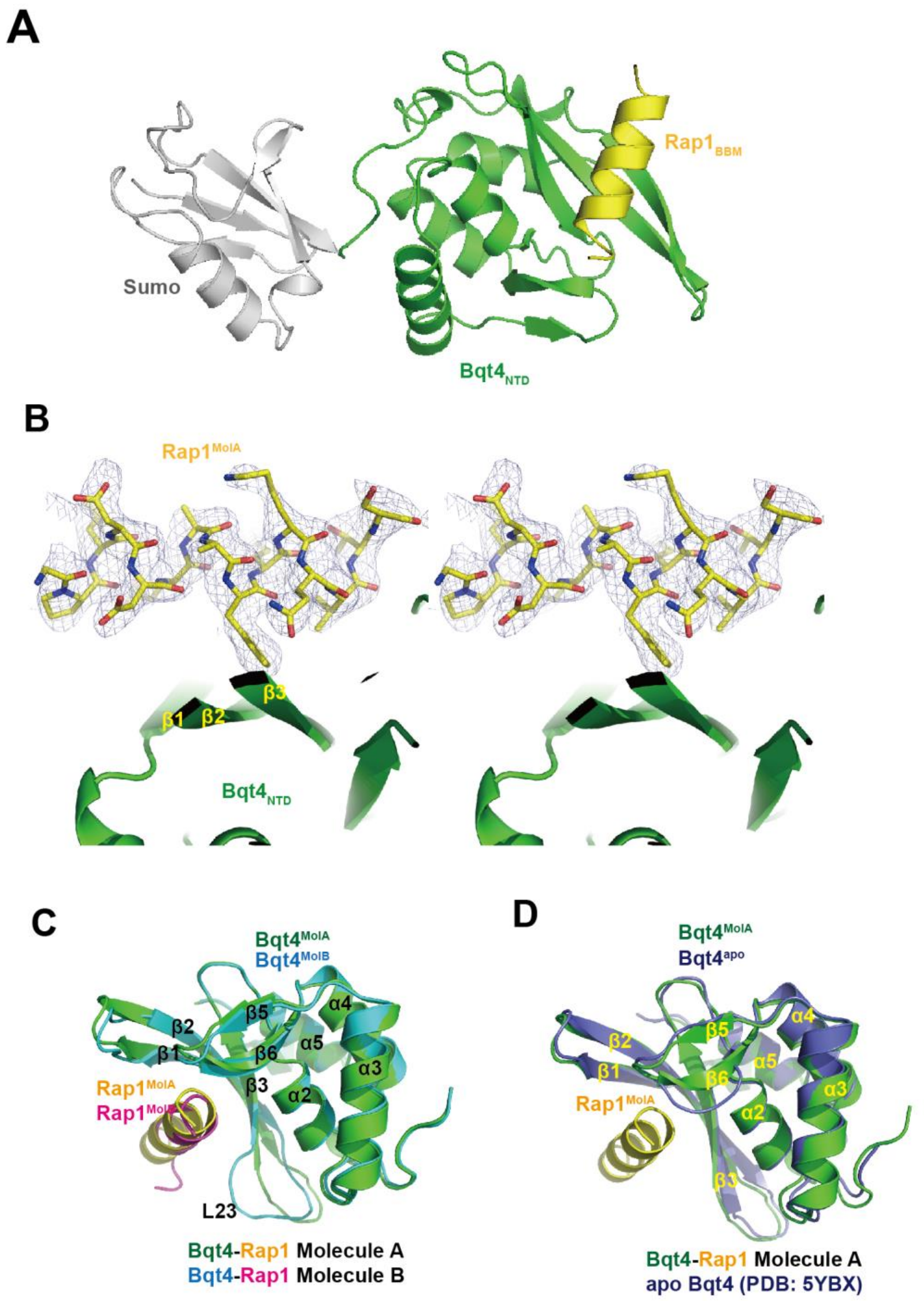

Supplementary Figure 2. The crystal structure of Sumo-fused Bqt4$_{NTD}$ in complex with Rap1$_{BBM}$.

A. The overall complex structure is shown in cartoon model. Sumo, grey; Bqt4$_{NTD}$, green; Rap1$_{BBM}$, yellow.

B. Stereo view of the electron density (2Fo-Fc) map that shows residues of $Rap1_{BBM}$ are ordered in the crystal. Refined model of $Rap1_{BBM}$ is shown in the density map. Contour is drawn at 1δ.

C. Superimposition of two $Bqt4_{NTD}$-$Rap1_{BBM}$ complexes in one asymmetrical unit. These two complexes have minor conformational differences in some loops, particularly the loop (L23) between β2 and β3 of Bqt4. The N-terminal extensions of $Rap1_{BBM}$ also subtly vary.

D. Superimposition of apo $Bqt4_{NTD}$ (PDB: 5YBX) and $Bqt4_{NTD}$-$Rap1_{BBM}$ complex shows that Rap1-binding induces formation of β5 and β6 of Bqt4.

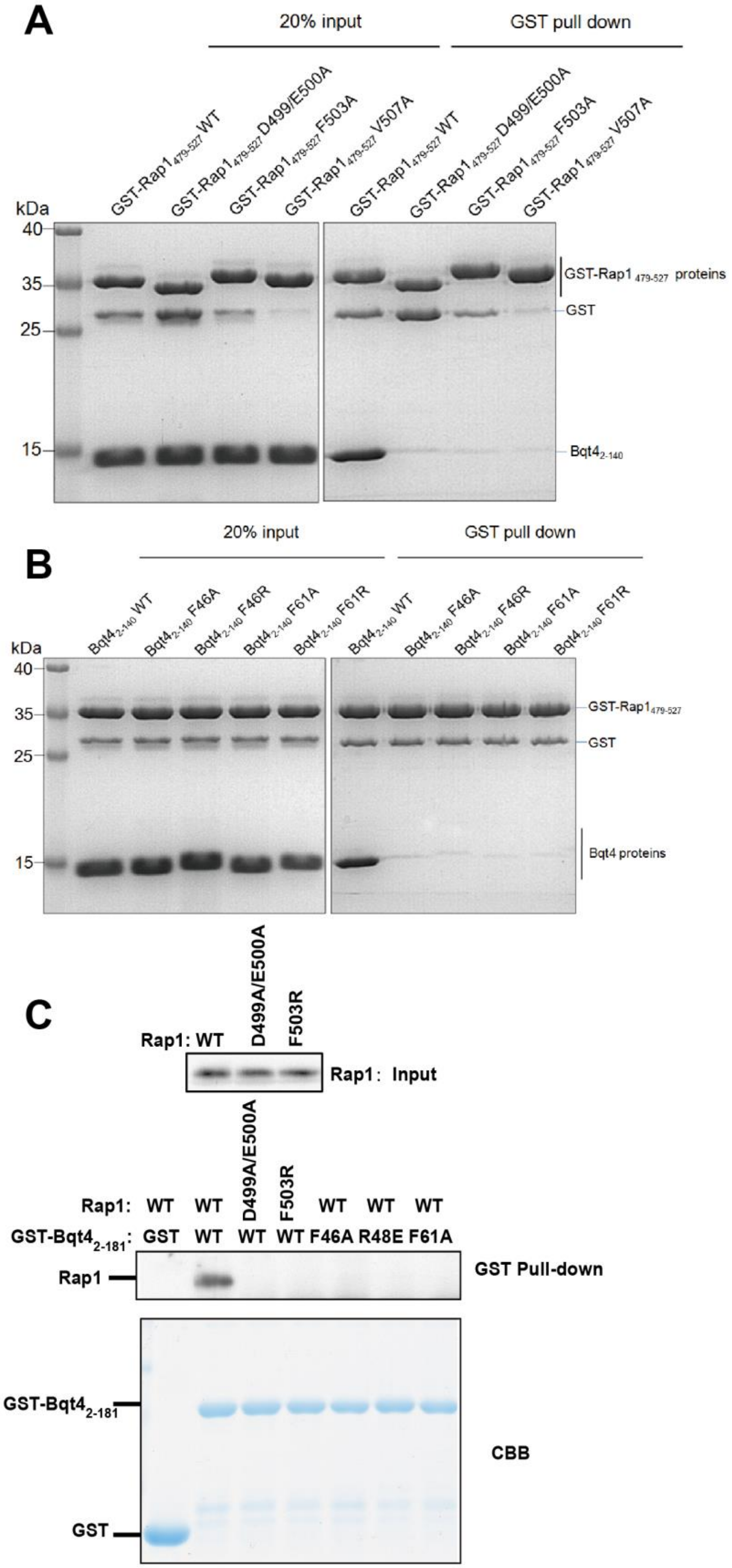

A
20% input
GST pull down
GST-Rap1 479-527 WT
GST-Rap1 479-527 D499/E500A
GST-Rap1 479-527 F503A
GST-Rap1 479-527 V507A
kDa
40
35
25
15
GST-Rap1 479-527 proteins
GST
Bqt4 2-140
B
Bqt4 2-140 WT
Bqt4 2-140 F46A
Bqt4 2-140 F46R
Bqt4 2-140 F61A
Bqt4 2-140 F61R
GST-Rap1 479-527
Bqt4 proteins
C
Rap1: WT D499A/E500A F503R
Rap1 : Input
Rap1: WT WT D499A/E500A F503R WT WT WT
GST-Bqt4 2-181: GST WT WT WT F46A R48E F61A
Rap1
GST Pull-down
GST-Bqt4 2-181
GST
CBB

Supplementary Figure 3. Mutational analyses of Bqt4-Rap1 complex.

A. GST pull-down assays showed that mutations of interface residues of Rap1 disrupted the binding to $Bqt4_{2-140}$. Purified GST-fused $Rap1_{479-527}$ and Rap1 mutants were used to pull down $Bqt4_{2-140}$. The input controls were shown in the left panel and GST pull-down samples were shown in the right panel.

B. GST pull-down assays showed that mutations on Bqt4 disrupted the binding to $Rap1_{479-527}$. Purified GST-fused $Rap1_{479-527}$ was used to pull down $Bqt4_{2-140}$ or Bqt4 mutants.

C. GST Pull-down assays using GST-$Bqt4_{2-181}$ with various mutations and yeast cell lysates expressing the wild-type Rap1, $Rap1^{D499A/E500A}$ or $Rap1^{F503R}$. The top panel showed the Rap1 input of cell lysates. The middle panel showed the immuno-blotting of Rap1 after pull-down. The bottom panel showed Coomassie Brilliant Blue (CBB) staining of GST proteins used for the pull-down assays.

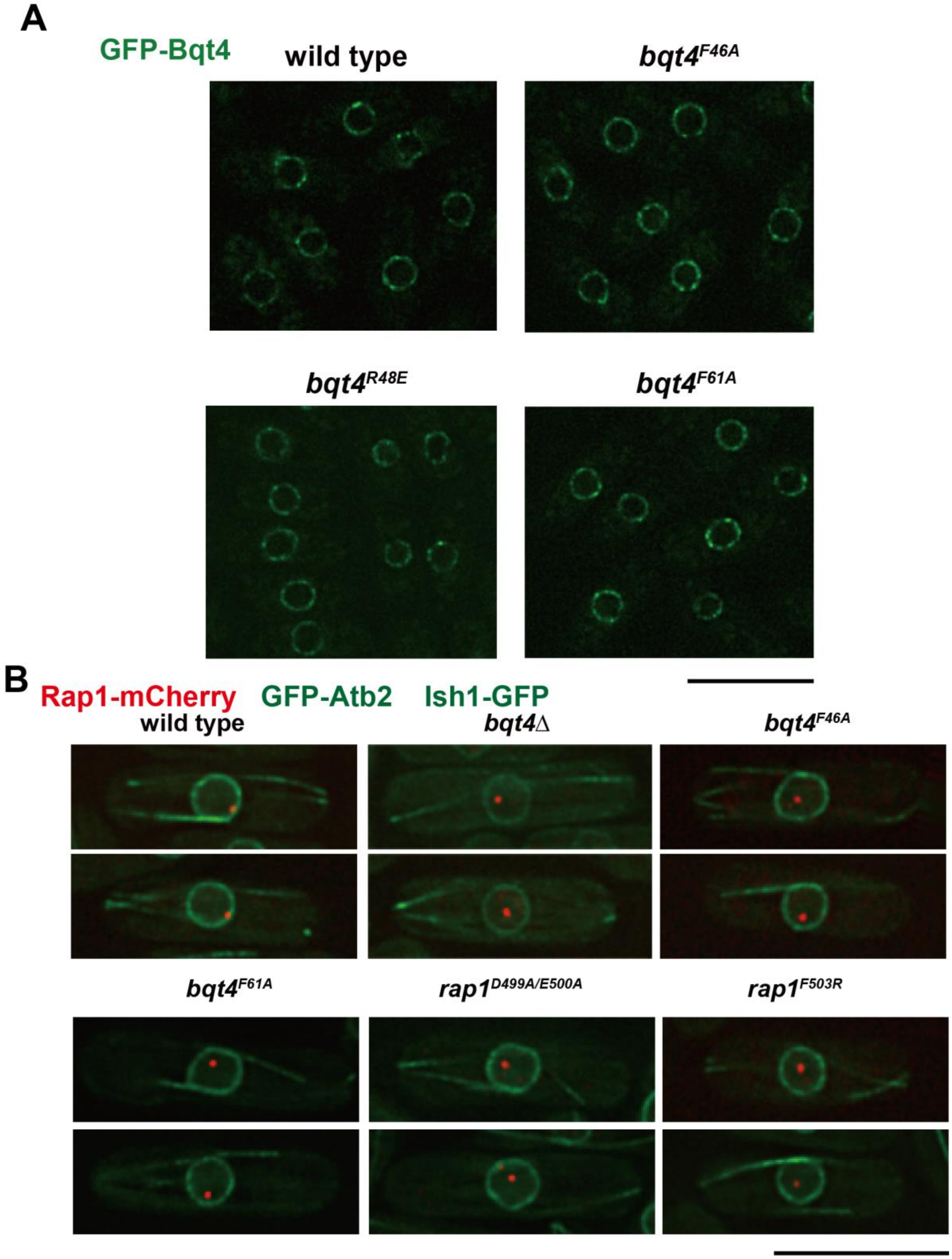

Supplementary Figure 4. Subcellular localizations of Bqt4 and Rap1.

A. Effects of Bqt4 mutations on localization of GFP-Bqt4 in living cells. GFP-Bqt4 showed exclusive localization to the NE in all Bqt4 wild type and mutant strains. The scale bars indicates 10 micrometer.

B. Effects of Bqt4 and Rap1 mutations on localization of Rap1 as shown by microscopic observation of living cells expressing Rap1-mCherry at 32°C in YES medium. The NE and microtubules were visualized by Ish1-GFP and GFP-Atb2, respectively. Each image shows a section of the nuclear mid-plane. The scale bars indicates 10 micrometer.

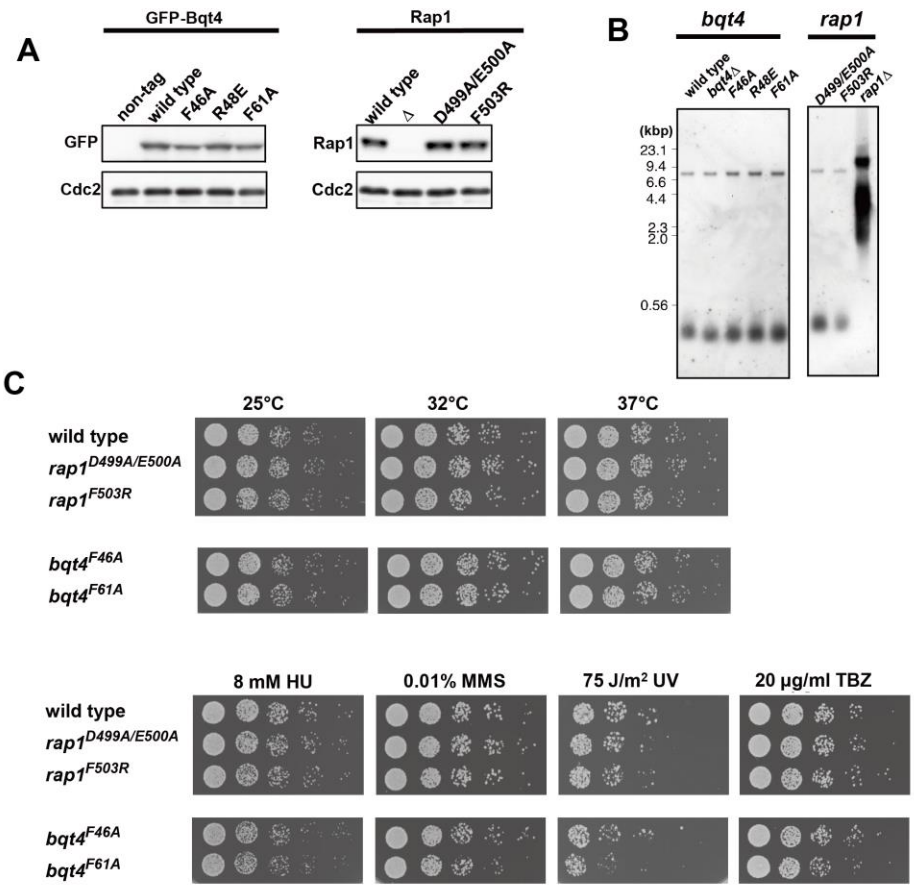

Supplementary Figure 5. Functional analyses of mutations on Bqt4 and Rap1.

A. Protein expression of each mutant form of GFP-Bqt4 (left) and Rap1 (right). Cdc2 was shown as a loading control.

B. Telomere DNA length in the *bqt4* and *rap1* mutant strains. Genomic DNA was digested with Apa1 and probed with telomere repeats.

C. Cell growth assays on YES plates at various temperatures, or on YES plates containing hydroxyurea (HU), methyl methanesulfonate (MMS), or thiabendazole (TBZ) at the indicated concentrations at 32°C. For the ultraviolet (UV) sensitivity assay, cells were subjected to UV irradiation immediately after spotting onto YES plates.

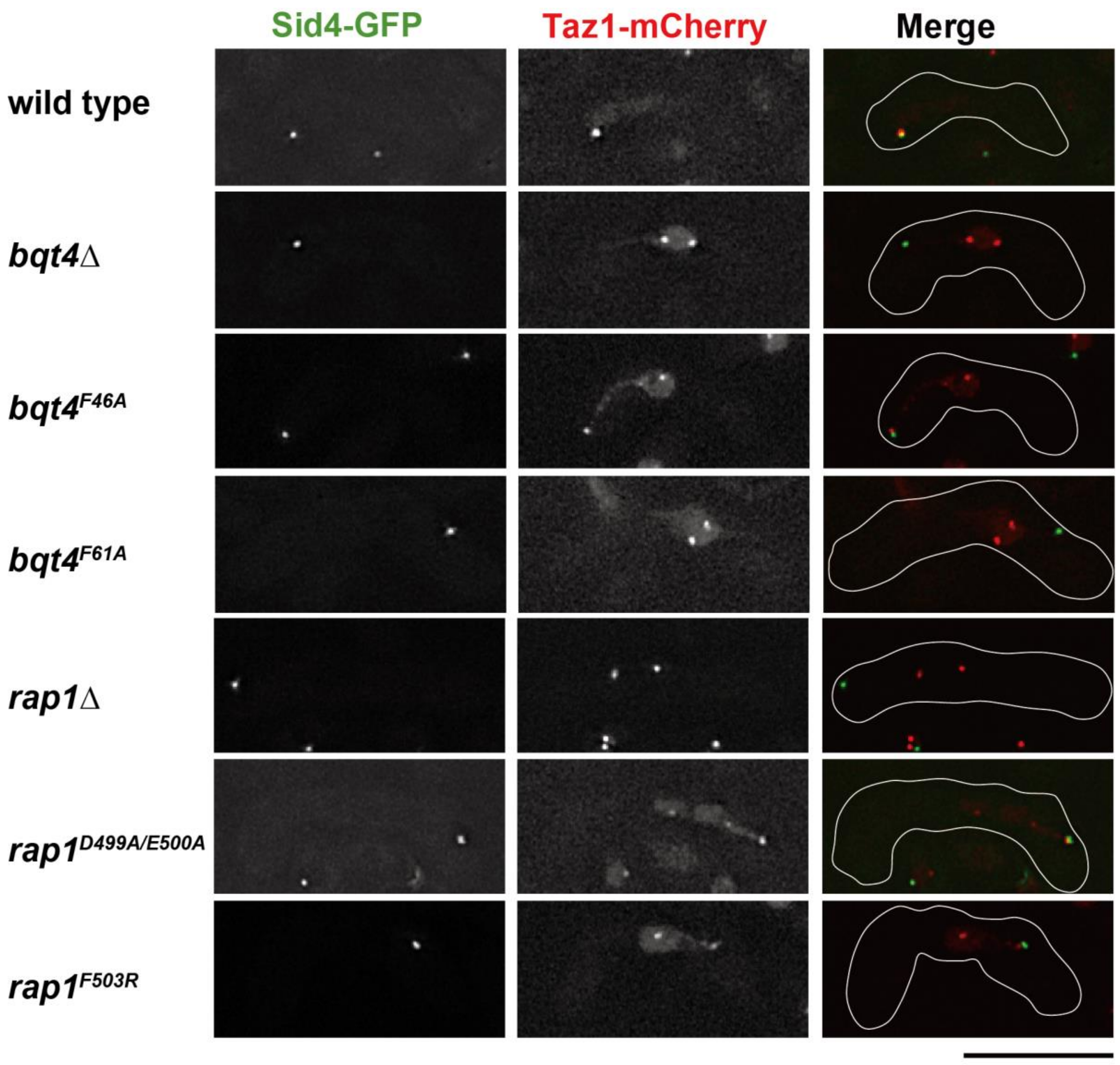

Supplementary Figure 6. Meiotic telomere clustering was defective in the *bqt4* and *rap1* mutants. Meiotic telomere clustering towards SPB was shown in three-dimensional deconvoluted images of a single cell. Homothallic haploid cells were incubated on MEA plates at 28°C for 10 hrs. Telomeres and SPB were visualized with Taz1-mCherry and Sid4-GFP, respectively. The scale bars indicates 10 micrometer.

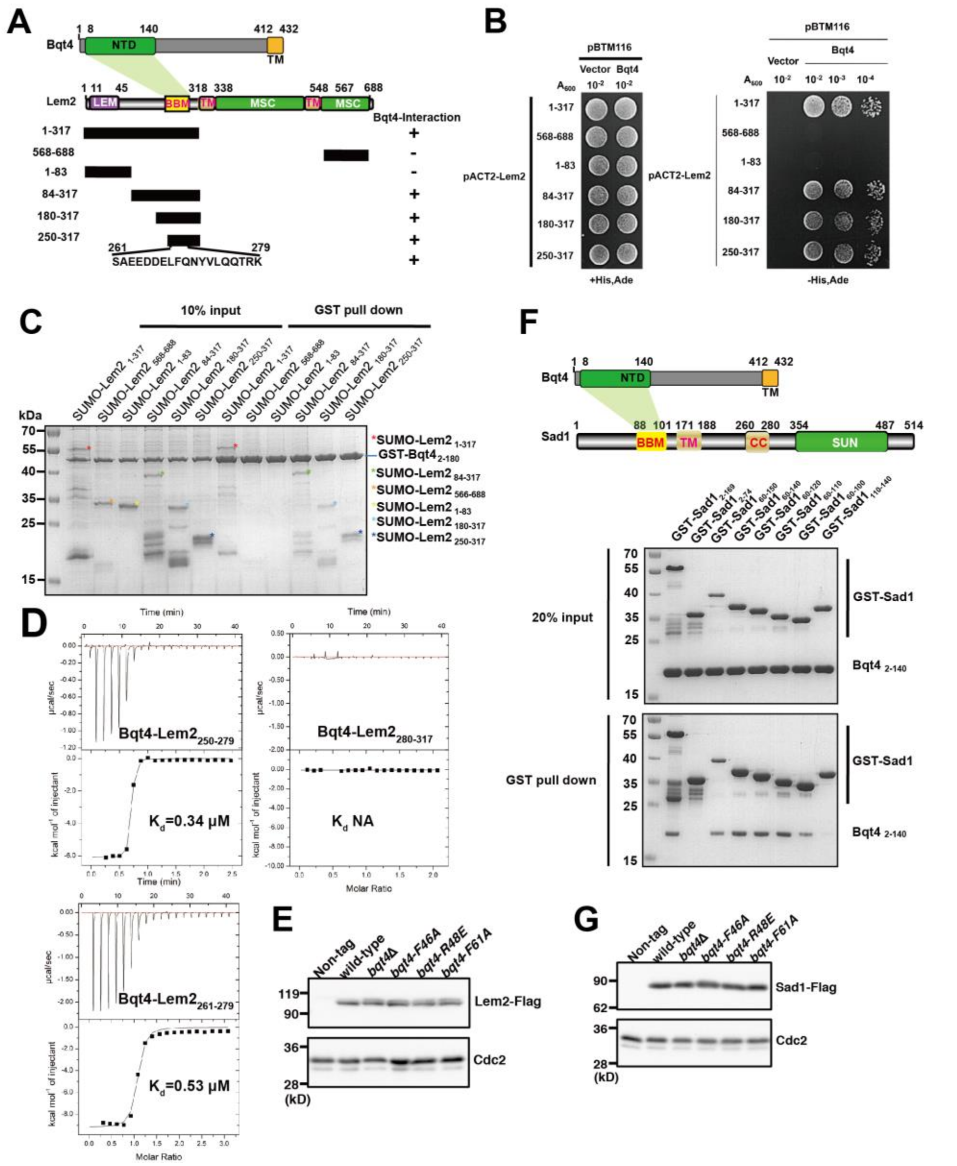

Supplementary Figure 7. Mapping the interaction regions in Bqt4-Lem2 and Bqt4-Sad1 complexes.

A, Summary of interactions between Bqt4 and Lem2. Domains in Bqt4 and Lem2: NTD, Bqt4 N-Terminal Domain; TM, Transmembrane helix; LEM, LAP2-Emerin-MAN1 domain; BBM, Bqt4-Binding Motif; MSC, MAN1/Src1 C-terminal domain. The Lem2 fragments with Bqt4-binding ability were labeled with “+”, and Lem2 fragments not binding Bqt4 were labeled with “-”.

B, Mapping the interaction domains between Bqt4 and Lem2 by yeast two-hybrid assays. $Lem2_{250-317}$ was identified to be the minimum fragment binding to Bqt4.

C, Mapping the interaction domains between Bqt4 and Lem2 by GST pull-down assays. GST-$Bqt4_{2-180}$ was used to pull down different fragments of Lem2 fused with SUMO tag. The input controls were shown as 10% of total proteins used for pull down assays. GST pull-down samples were eluted by SDS-PAGE loading buffer.

D, Mapping the interaction domains between Bqt4 and Lem2 by ITC assays. The minimum fragment $Lem2_{250-317}$ identified from yeast two-hybrid and GST pull-down assays was further divided into two fragments (250-279 and 280-317). The $Lem2_{250-279}$ with Bqt4-binding activity was further refined to $Lem2_{261-279}$ with similar Bqt4-binding activity.

E. Protein levels of Lem2-Flag in the presence of Bqt4 mutations. Cdc2 was shown as a loading control. Bqt4 mutations have no effect on expression level and stability of Lem2 proteins *in vivo*.

F. Mapping the interaction domains between Bqt4 and Sad1 by GST pull-down assays. Different fragments of Sad1 were fused with GST and used to pull down $Bqt4_{2-140}$. $Sad1_{60-100}$ was the minimum fragment with Bqt4-binding activity. This fragment contained a sequence motif sharing striking similarity with Bqt4-binding-motifs from Rap1 and Sad1.

G. Protein levels of Sad1-Flag in the presence of Bqt4 mutations. Cdc2 was shown as a loading control. Bqt4 mutations have no effect on expression level and stability of Sad1 proteins *in vivo*.

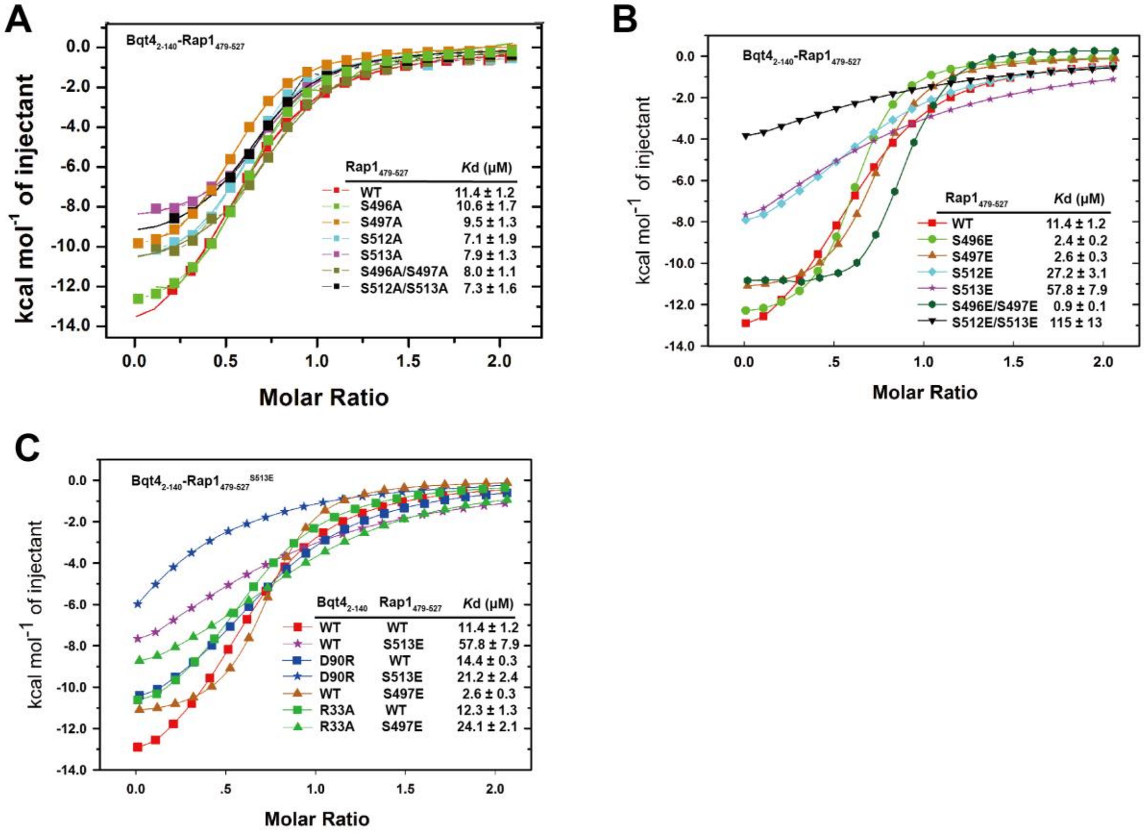

Supplementary Figure 8. ITC analyses of Bqt4-Rap1 interactions.

A. ITC measurements of interactions between $Bqt4_{NTD}$ and $Rap1_{479-527}$ with serine-to-alanine mutations. The fitting curves of the titration data and the derived dissociation constant (Kd) value ± SD were shown. Serine residues on Rap1 per se have very little direct contacts with $Bqt4_{NTD}$. The ITC curve for the wild type $Bqt4_{NTD}$-$Rap1_{479-527}$ was the same one used in Figure 2C and 2D as a control, and also used in the following panel B and C.

B. ITC measurements of interactions between $Bqt4_{NTD}$ and $Rap1_{479-527}$ with serine-to-glutamate mutations. These phosphorylation-mimic mutations on the C-terminus of $Rap1_{BBM}$ decreased Bqt4-Rap1 interaction, while phosphorylation-mimic mutations on the N-terminus of $Rap1_{BBM}$ increased Bqt4-Rap1 interaction.

C. Effects of Bqt4 mutation on the $Bqt4_{NTD}$-$Rap1_{479\text{-}527}$ interactions. Some Bqt4 residues may sense the phosphorylation states of Rap1. Bqt4 D90 may repulse the C-terminal phosphorylation of Rap1, and $Bqt4^{D90R}$ mutation increased the binding affinity with $Rap1^{S513E}$ compared with $Bqt4^{WT}$-$Rap1^{S513}$. Bqt4 R33 may form new ionic contacts with N-terminal phosphorylation of Rap1, and $Bqt4^{R33A}$ decreased the interaction with $Rap1^{S497E}$.

Supplementary Table 1. *S. pombe* strains used in this study

Figure 2E and Supplementary Figure 4B

JP4539	*h*$^{+}$ *ade6-M210 leu1-32 ura4-D18 his2-245 GFP-atb2*::*lys1*$^{+}$ *ish1-HA-GFP*::*kanMX rap1-mCherry*::*hphMX*
JP4610	*h*$^{+}$ *ade6-M210 leu1-32 ura4-D18 his2-245 GFP-atb2*::*lys1*$^{+}$ *ish1-HA-GFP*::*kanMX rap1-mCherry*::*hphMX bqt4*::*ura4*$^{+}$
JP4652	*h*$^{+}$ *ade6-M210 leu1-32 ura4-D18 his2-245 GFP-atb2*::*lys1*$^{+}$ *ish1-HA-GFP*::*kanMX rap1-mCherry*::*hphMX bqt4*^{F46A}
JP4654	*h*$^{+}$ *ade6-M210 leu1-32 ura4-D18 his2-245 GFP-atb2*::*lys1*$^{+}$ *ish1-HA-GFP*::*kanMX rap1-mCherry*::*hphMX bqt4*^{F61A}
JP4718	*h*$^{+}$ *ade6-M210 leu1-32 ura4-D18 his2-245 GFP-atb2*::*lys1*$^{+}$ *ish1-HA-GFP*::*kanMX rap1-mCherry*::*hphMX rap1*$^{D499A/E500A}$
JP4715	*h*$^{+}$ *ade6-M210 leu1-32 ura4-D18 his2-245 GFP-atb2*::*lys1*$^{+}$ *ish1-HA-GFP*::*kanMX rap1-mCherry*::*hphMX rap1*^{F503R}

Figure 3A

JK81	*h*$^{+}$ *ade6-M216 leu1-32 ura4-D18 his2-245 taz1-mCherry*::*kanMX ish1-HA-GFP*::*kanMX lys1*$^{+}$::*nda3p-GFP-atb2*
JP842	*h*$^{+}$ *ade6-M216 leu1-32 ura4-D18 his2-245 taz1-mCherry*::*kanMX ish1-HA-GFP*::*kanMX lys1*$^{+}$::*nda3p-GFP-atb2 bqt4*::*ura4*$^{+}$
JP4648	*h*$^{+}$ *ade6-M216 leu1-32 ura4-D18 his2-245 taz1-mCherry*::*kanMX ish1-HA-GFP*::*kanMX lys1*$^{+}$::*nda3p-GFP-atb2 bqt4*^{F46A}
JP4650	*h*$^{+}$ *ade6-M216 leu1-32 ura4-D18 his2-245 taz1-mCherry*::*kanMX ish1-HA-GFP*::*kanMX lys1*$^{+}$::*nda3p-GFP-atb2 bqt4*^{F61A}
JP836	*h*$^{+}$ *ade6-M216 leu1-32 ura4-D18 his2-245 taz1-mCherry*::*kanMX ish1-HA-GFP*::*kanMX lys1*$^{+}$::*nda3p-GFP-atb2 rap1*::*ura4*$^{+}$
JP4680	*h*$^{+}$ *ade6-M216 leu1-32 ura4-D18 his2-245 taz1-mCherry*::*kanMX ish1-HA-GFP*::*kanMX lys1*$^{+}$::*nda3p-GFP-atb2 rap1*$^{D499A/E500A}$
JP4678	*h*$^{+}$ *ade6-M216 leu1-32 ura4-D18 his2-245 taz1-mCherry*::*kanMX ish1-HA-GFP*::*kanMX lys1*$^{+}$::*nda3p-GFP-atb2 rap1*^{F503R}

Figure 3B and Supplementary Figure 6

JP4890	*h*^{90} *ade6-M210 leu1-32 ura4-D18 sid4-GFP*$^{+}$::*kanMX taz1-mCherry*::*hphMX*
JP4892	*h*^{90} *ade6-M210 leu1-32 ura4-D18 sid4-GFP*$^{+}$::*kanMX taz1-mCherry*::*hphMX bqt4*::*ura4*$^{+}$
JP5205	*h*^{90} *ade6-M210 leu1-32 ura4-D18 sid4-GFP*$^{+}$::*kanMX taz1-mCherry*::*hphMX bqt4*^{F46A}
JP5207	*h*^{90} *ade6-M210 leu1-32 ura4-D18 sid4-GFP*$^{+}$::*kanMX taz1-mCherry*::*hphMX bqt4*^{F61A}
JP5204	*h*^{90} *ade6-M210 leu1-32 ura4-D18 sid4-GFP*$^{+}$:: *hphMX taz1-mCherry*:: *kanMX rap1*::*ura4*$^{+}$
JP5211	*h*^{90} *ade6-M210 leu1-32 ura4-D18 sid4-GFP*$^{+}$:: *hphMX taz1-mCherry*:: *kanMX rap1*$^{D499A/E500A}$
JP5213	*h*^{90} *ade6-M210 leu1-32 ura4-D18 sid4-GFP*$^{+}$:: *hphMX taz1-mCherry*:: *kanMX rap1*^{F503R}

Figures 3C

JP245	*h*^{90} *ade6-M210 leu1-32 ura4-D18*
JP839	*h*^{90} *ade6-M210 leu1-32 ura4-D18 bqt4*::*ura4*$^{+}$

JP4606	*h*^{90} *ade6-M210 leu1-32 ura4-D18 bqt4*^{F46A}
JP4608	*h*^{90} *ade6-M210 leu1-32 ura4-D18 bqt4*^{F61A}
JP3743	*h*^{90} *ade6-M210 leu1-32 ura4-D18 rap1*::*ura4*$^{+}$
JP4676	*h*^{90} *ade6-M210 leu1-32 ura4-D18 rap1*$^{D499A/E500A}$
JP4675	*h*^{90} *ade6-M210 leu1-32 ura4-D18 rap1*^{F503R}

Supplementary Figure 3C

JP245	*h*^{90} *ade6-M210 leu1-32 ura4-D18*
JP4676	*h*^{90} *ade6-M210 leu1-32 ura4-D18 rap1*$^{D499A/E500A}$
JP4675	*h*^{90} *ade6-M210 leu1-32 ura4-D18 rap1*^{F503R}

Supplementary Figure 4A

JP4459	*h*^{90} *ade6-M210 leu1-32 ura4-D18 GFP-bqt4*
JP4697	*h*^{90} *ade6-M210 leu1-32 ura4-D18 GFP-bqt4*^{F46A}
JP4513	*h*^{90} *ade6-M210 leu1-32 ura4-D18 GFP-bqt4*^{R48E}
JP4699	*h*^{90} *ade6-M210 leu1-32 ura4-D18 GFP-bqt4*^{F61A}

Supplementary Figure 5A

JP245	*h*^{90} *ade6-M210 leu1-32 ura4-D18*
JP4459	*h*^{90} *ade6-M210 leu1-32 ura4-D18 GFP-bqt4*
JP4697	*h*^{90} *ade6-M210 leu1-32 ura4-D18 GFP-bqt4*^{F46A}
JP4513	*h*^{90} *ade6-M210 leu1-32 ura4-D18 GFP-bqt4*^{R48E}
JP4699	*h*^{90} *ade6-M210 leu1-32 ura4-D18 GFP-bqt4*^{F61A}

JP245	*h*^{90} *ade6-M210 leu1-32 ura4-D18*
JP3743	*h*^{90} *ade6-M210 leu1-32 ura4-D18 rap1*::*ura4*$^{+}$
JP4676	*h*^{90} *ade6-M210 leu1-32 ura4-D18 rap1*$^{D499A/E500A}$
JP4675	*h*^{90} *ade6-M210 leu1-32 ura4-D18 rap1*^{F503R}

Supplementary Figure 5B

JP245	*h*^{90} *ade6-M210 leu1-32 ura4-D18*
JP839	*h*^{90} *ade6-M210 leu1-32 ura4-D18 bqt4*::*ura4*$^{+}$
JP4606	*h*^{90} *ade6-M210 leu1-32 ura4-D18 bqt4*^{F46A}
JP4359	*h*^{90} *ade6-M210 leu1-32 ura4-D18 bqt4*^{R48E}
JP4608	*h*^{90} *ade6-M210 leu1-32 ura4-D18 bqt4*^{F61A}
JP4676	*h*^{90} *ade6-M210 leu1-32 ura4-D18 rap1*$^{D499A/E500A}$
JP4675	*h*^{90} *ade6-M210 leu1-32 ura4-D18 rap1*^{F503R}
JP3743	*h*^{90} *ade6-M210 leu1-32 ura4-D18 rap1*::*ura4*$^{+}$

JP245	*h*^{90} *ade6-M210 leu1-32 ura4-D18*
JP839	*h*^{90} *ade6-M210 leu1-32 ura4-D18 bqt4*::*ura4*$^{+}$
JP4835	*h*^{90} *ade6-M210 leu1-32 ura4-D18 GFP-bqt4-3E-A*
JP4837	*h*^{90} *ade6-M210 leu1-32 ura4-D18 GFP-bqt4-3E-B*

Supplementary Figure 5C

JP245	*h*^{90} *ade6-M210 leu1-32 ura4-D18*
JP4676	*h*^{90} *ade6-M210 leu1-32 ura4-D18 rap1*$^{D499A/E500A}$
JP4675	*h*^{90} *ade6-M210 leu1-32 ura4-D18 rap1*^{F503R}
JP4606	*h*^{90} *ade6-M210 leu1-32 ura4-D18 bqt4*^{F46A}
JP4608	*h*^{90} *ade6-M210 leu1-32 ura4-D18 bqt4*^{F61A}

Supplementary Figure 7E

JP5624	*h^{90} ade6-M210 leu1-32 ura4-D18 lem2-Flag::kanMX*
JP5625	*h^{90} ade6-M210 leu1-32 ura4-D18 lem2-Flag::kanMX bqt4::$ura4^{+}$*
JP5626	*h^{90} ade6-M210 leu1-32 ura4-D18 lem2-Flag::kanMX $bqt4^{F46A}$*
JP5627	*h^{90} ade6-M210 leu1-32 ura4-D18 lem2-Flag::kanMX $bqt4^{R48E}$*
JP5628	*h^{90} ade6-M210 leu1-32 ura4-D18 lem2-Flag::kanMX $bqt4^{F61A}$*

Supplementary Figure 7G

JP5619	*h^{90} ade6-M210 leu1-32 ura4-D18 sad1-Flag::kanMX*
JP5620	*h^{90} ade6-M210 leu1-32 ura4-D18 sad1-Flag::kanMX bqt4::$ura4^{+}$*
JP5621	*h^{90} ade6-M210 leu1-32 ura4-D18 sad1-Flag::kanMX $bqt4^{F46A}$*
JP5622	*h^{90} ade6-M210 leu1-32 ura4-D18 sad1-Flag::kanMX $bqt4^{R48E}$*
JP5623	*h^{90} ade6-M210 leu1-32 ura4-D18 sad1-Flag::kanMX $bqt4^{F61A}$*
